# Determining cardiovascular risk in patients with unattributed chest pain in UK primary care: an electronic health record study

**DOI:** 10.1093/eurjpc/zwad055

**Published:** 2023-03-10

**Authors:** Kelvin P Jordan, Trishna Rathod-Mistry, Danielle A van der Windt, James Bailey, Ying Chen, Lorna Clarson, Spiros Denaxas, Richard A Hayward, Harry Hemingway, Theocharis Kyriacou, Mamas A Mamas

**Affiliations:** School of Medicine, David Weatherall Building, University Road, Keele University, Staffordshire ST5 5BG, UK; School of Medicine, David Weatherall Building, University Road, Keele University, Staffordshire ST5 5BG, UK; Centre for Statistics in Medicine, Nuffield Department of Orthopaedics, Rheumatology, and Musculoskeletal Sciences, University of Oxford, Windmill Road, Oxford OX3 7LD, UK; School of Medicine, David Weatherall Building, University Road, Keele University, Staffordshire ST5 5BG, UK; School of Medicine, David Weatherall Building, University Road, Keele University, Staffordshire ST5 5BG, UK; School of Medicine, David Weatherall Building, University Road, Keele University, Staffordshire ST5 5BG, UK; Wisdom Lake Academy of Pharmacy, Xi'an Jiaotong-Liverpool University, Suzhou 215123, Jiangsu, China; School of Medicine, David Weatherall Building, University Road, Keele University, Staffordshire ST5 5BG, UK; Institute of Health Informatics, University College London, 222 Euston Road, London NW1 2DA, UK; Health Data Research UK, University College London, 222 Euston Road, London NW1 2DA, UK; School of Medicine, David Weatherall Building, University Road, Keele University, Staffordshire ST5 5BG, UK; Institute of Health Informatics, University College London, 222 Euston Road, London NW1 2DA, UK; The National Institute for Health Research University College London Hospitals Biomedical Research Centre, Maple House 1st floor, 149 Tottenham Court Road, London W1T 7DN, UK; School of Computing and Mathematics, Keele University, Staffordshire ST5 5AA, UK; Keele Cardiovascular Research Group, School of Medicine, David Weatherall Building, University Road, Keele University, Staffordshire ST5 5BG, UK

**Keywords:** Chest pain, Cardiovascular disease, Primary health care, Risk, Electronic health records, Epidemiology

## Abstract

**Aims:**

Most adults presenting in primary care with chest pain symptoms will not receive a diagnosis (‘unattributed’ chest pain) but are at increased risk of cardiovascular events. To assess within patients with unattributed chest pain, risk factors for cardiovascular events and whether those at greatest risk of cardiovascular disease can be ascertained by an existing general population risk prediction model or by development of a new model.

**Methods and results:**

The study used UK primary care electronic health records from the Clinical Practice Research Datalink linked to admitted hospitalizations. Study population was patients aged 18 plus with recorded unattributed chest pain 2002–2018. Cardiovascular risk prediction models were developed with external validation and comparison of performance to QRISK3, a general population risk prediction model. There were 374 917 patients with unattributed chest pain in the development data set. The strongest risk factors for cardiovascular disease included diabetes, atrial fibrillation, and hypertension. Risk was increased in males, patients of Asian ethnicity, those in more deprived areas, obese patients, and smokers. The final developed model had good predictive performance (external validation c-statistic 0.81, calibration slope 1.02). A model using a subset of key risk factors for cardiovascular disease gave nearly identical performance. QRISK3 underestimated cardiovascular risk.

**Conclusion:**

Patients presenting with unattributed chest pain are at increased risk of cardiovascular events. It is feasible to accurately estimate individual risk using routinely recorded information in the primary care record, focusing on a small number of risk factors. Patients at highest risk could be targeted for preventative measures.

## Graphical Abstract

**Graphical Abstract:**
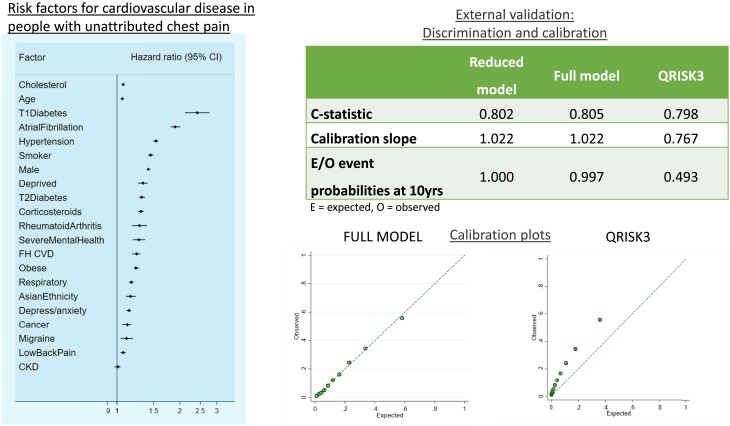

## Introduction

Chest pain is a common symptom for patients in primary care. In the UK, around 2% of adults will present in primary care with chest pain symptoms annually.^1–4^ Whilst general practitioners (GPs) may pursue investigations and diagnose angina or a non-cardiac causes (such as gastro-oesophageal disease, musculoskeletal disease, or anxiety^5^), many patients do not receive a specific diagnosis.^1,2,6^ Patients with such unattributed chest pain have an increased risk of future cardiovascular events compared to those without chest pain,^7,8^ and to patients diagnosed with non-coronary causes.^4,9^ However, the majority of patients with unattributed chest pain do not receive preventative medication (for example, lipid-lowering drugs), even those at potentially higher risk of cardiovascular disease.^9^

Identification of those who have the greatest risk of future cardiovascular events would allow targeting of key modifiable cardiovascular risk factors with preventative management.^6^ Cardiovascular disease risk algorithms exist for the general population but may have less validity in other populations. The QRISK score (the most recent being QRISK3^10^) is the recommended algorithm for assessing cardiovascular risk by the UK National Institute for Health and Care Excellence (NICE)^11^ and was developed and validated for use in UK primary care to estimate the risk of cardiovascular events over ten years in patients known to be currently free of cardiovascular disease and not currently prescribed lipid lowering medication. However, QRISK3 was developed and validated in the general population and may not be valid for use in patients with unattributed chest pain, some of whom are already being prescribed lipid-lowering medication and who have a higher underlying risk of cardiovascular disease than the general population.

The aim of this study was to assess, within patients presenting to primary care with chest pain for which no diagnosis is given, whether those at greatest risk of cardiovascular disease and hence for whom early preventative medication and targeting of key risk factors may be most beneficial, can be accurately identified. Setting the study within routinely recorded EHR ensured identified risk factors are readily available to GPs. The specific objectives were (i) to assess the performance of a general population risk prediction model (QRISK3) in this population, (ii) determine key risk factors for cardiovascular disease in patients with unattributed chest pain in UK primary care, and (iii) derive and validate improved prediction models for cardiovascular disease in these patients.

## Methods

### Setting

The study was set within the Clinical Practice Research Datalink (CPRD). All analyses were performed using the CPRD Aurum database with validation performed in the CPRD GOLD database. Aurum is a UK primary care EHR database containing anonymized information routinely recorded in (as of November 2021) over 1400 general practices (over 40 million patients) which use EMIS Web® software.^12,13^ The CPRD GOLD database includes information from over 900 general practices which use Vision® software.^13,14^ Practices used in this study were the subgroup of English practices which have consented to linkage to inpatient diagnoses and procedures from Hospital Episode Statistics (HES), cause-specific mortality from the Office for National Statistics (ONS), and neighbourhood deprivation scores. Data linkage is undertaken by a trusted third party (NHS Digital) using an eight-stage deterministic methodology involving the National Health Service (NHS) number, gender, dob, and postcode.^15^ The majority of patients (for example, 96% for CPRD GOLD to HES in 2018) are matched on exact NHS number, gender, dob, and postcode, or exact NHS number, gender, and dob. The de-identified linked data are then sent to CPRD with the relevant requested anonymized data then sent on to researchers. UK primary care has traditionally used Read codes (up to 2018) to electronically record morbidities and symptoms presented by patients whilst more recently SNOMED-CT is used. UK secondary care uses ICD-10 and OPCS Classification of Interventions and Procedures codes (OPCS-4) to record morbidities and procedures, respectively. The study followed the PROGRESS framework for prognostic research^16,17^ and is reported using TRIPOD guidance.^18^

### Study population

As described previously,^9^ the study population was patients aged ≥18 years presenting to primary care between 2002 and 2018 with incident chest pain with no cause recorded. The date of incident chest pain was defined as the index date. Patients were excluded if they had cardiovascular disease recorded prior to or up to 6 months after their index date, a non-coronary cause (such as costochondritis) recorded for their chest pain at index date or in the 6 months after index date, <2 prior years of registration at their general practice, or <6 months of follow-up data after index date. We allowed 6 months after index date (the ‘diagnostic window’) for investigations and diagnosis related to initial presentation to occur.

Unattributed chest pain was defined using Read codes recorded in primary care for symptoms not clearly specifying the cause of the pain. This included codes with terms such as ‘chest pain not otherwise specified’ and ‘chest tightness’. Read code lists were derived through consensus work in a previous study^4^ and are shown in supplementary material online, *Table S1*.

### Outcomes

The primary outcome was incident cardiovascular disease (CVD) defined as a record of any of fatal or non-fatal acute myocardial infarction, angina, coronary heart disease not otherwise specified, heart failure, ventricular arrhythmia, cardiac arrest, ischaemic stroke, haemorrhagic stroke, stroke type not specified, transient ischaemic attack, peripheral arterial disease, abdominal aortic aneurysm, sudden cardiac death, percutaneous coronary intervention, and coronary artery bypass graft surgery. Outcomes were captured from the primary care, secondary care, and ONS death registry records, using derived and validated algorithms.^19^

Patients were followed from end of the 6-month diagnostic window until end of follow-up defined as the earliest of date of death, transfer out of practice, occurrence of outcome, or end of study (31 December 2018).

### Risk factors

The potential risk factors were decided by consensus of the study team by consideration of those included in the QRISK3 algorithm,^10^ and potential alternative explanations for chest pain and comorbidities previously suggested to be predictive of cardiovascular disease.^20,21^ These are listed in *Table 1*. Comorbidities were measured in the 24 months prior to index date up to end of the 6-month diagnostic window. Prescription-based comorbidities (treated hypertension, corticosteroids) were defined as at least two prescriptions in this 30-month period. Body mass index (BMI, categorized into underweight, normal, overweight, obese, and not recorded) and smoking status (never, current, ex, and not recorded) were based on record nearest, but prior to, the end of the 6-month diagnostic window. Body mass index was categorized to allow use of information captured by Read codes (for example, diagnosis codes for overweight or obese) where no BMI value was recorded. Neighbourhood deprivation was based on the Townsend score and categorized at the quintile scores. As cholesterol values were not recorded comprehensively and unlikely to be missing at random, we imputed total cholesterol/HDL ratio based on the mean value for those in the data set with the same age, gender, and ethnicity.

**Table 1:** Patient characteristics in development data set, *n* (%) unless stated

	Total	No CVD in follow-up	CVD in follow-up
*n*	374 917	331 966	42 951
Age: mean (SD)	47.8 (16.5)	45.9 (15.91)	61.9 (14.11)
Sex: female	199 607 (53.2)	178 188 (53.7)	21 419 (49.9)
Ethnicity			
White/not recorded	332 825 (88.8)	293 132 (88.3)	39 693 (92.4)
Asian	21 841 (5.8)	19 875 (6.0)	1966 (4.6)
Black	13 769 (3.7)	12 795 (3.9)	974 (2.3)
Other	6482 (1.7)	6164 (1.9)	318 (0.7)
Deprivation			
Least	81 263 (21.7)	71 983 (21.7)	9280 (21.6)
2nd	75 267 (20.1)	66 173 (19.9)	9094 (21.2)
3rd	71 353 (19.0)	62 863 (18.9)	8490 (19.8)
4th	67 448 (18.0)	59 805 (18.0)	7643 (17.8)
Most	79 586 (21.2)	71 142 (21.4)	8444 (19.7)
Smoking status			
Never	173 126 (46.2)	155 856 (47.0)	17 270 (40.2)
Current	105 738 (28.2)	93 669 (28.2)	12 069 (28.1)
Ex	85 621 (22.8)	73 196 (22.1)	12 425 (28.9)
Not recorded	10 432 (2.8)	9245 (2.8)	1187 (2.8)
Diabetes type 1	1345 (0.4)	1079 (0.3)	266 (0.6)
Diabetes type 2	20 444 (5.5)	15 774 (4.8)	4670 (10.9)
FH: angina/heart attack <60 yrs	19 080 (5.1)	16 569 (5.0)	2511 (5.8)
CKD stage 3–5	19 283 (5.1)	14 562 (4.4)	4721 (11.0)
Atrial fibrillation	4771 (1.3)	2753 (0.8)	2018 (4.7)
Treated hypertension	77 639 (20.7)	58 551 (17.6)	19 088 (44.4)
Migraine	11 177 (3.0)	10 268 (3.1)	909 (2.1)
Rheumatoid arthritis	2362 (0.6)	1860 (0.6)	502 (1.2)
Severe mental illness	6968 (1.9)	5983 (1.8)	985 (2.3)
Corticosteroid medication	20 150 (5.4)	15 991 (4.8)	4159 (9.7)
Cholesterol/HDL ratio: mean (SD)	3.9 (1.0)	3.9 (1.0)	4.0 (1.1)
Body mass index			
Normal/underweight	130 030 (34.7)	117 862 (35.5)	12 168 (28.3)
Overweight	109 403 (29.2)	95 322 (28.7)	14 081 (32.8)
Obese	80 198 (21.4)	68 978 (20.8)	11 220 (26.1)
Not recorded	55 286 (14.7)	49 804 (15.0)	5482 (12.8)
Depression/anxiety	61 640 (16.4)	55 159 (16.6)	6481 (15.1)
Oesophageal reflux	35 095 (9.4)	29 931 (9.0)	5164 (12.0)
Respiratory	78 752 (21.0)	66 772 (20.1)	11 980 (27.9)
Osteoarthritis	18 425 (4.9)	13 935 (4.2)	4490 (10.5)
Low back pain	64 716 (17.3)	56 513 (17.0)	8203 (19.1)
Neck pain	25 857 (6.9)	22 333 (6.7)	3524 (8.2)
Cancer	9521 (2.5)	7744 (2.3)	1777 (4.1)
CVD in follow-up	42 951 (11.5) 19.3/1000py	—	—

Missing data cholesterol 49%, ethnicity 6%.

BP, blood pressure; CVD, cardiovascular disease; SD, standard deviation; py, person-year.

### Statistical analysis

#### Performance of QRISK3

The QRISK3 estimated 10-year CVD risk was calculated using the online open access gender-specific algorithms,^22^ replicated for use in Stata/MP 15.1 for Windows, and compared for different combinations of risk factors to the estimated risk produced by the online calculator. Determination of QRISK3 score requires actual BMI value; therefore, for patients with a recorded BMI category (underweight, normal, overweight, or obese) but no BMI value recorded, we allocated mean BMI value for those of the same BMI category, age, gender, and ethnicity. If there was no BMI category recorded, they were allocated the mean BMI value for those of the same age, gender, and ethnicity, as this is how missing data are imputed by QRISK3. For smoking, if the record only indicated current smoker and no evidence of level, then they were allocated the most frequent level of smoking (light, moderate, and heavy) for current smokers of their age, gender, and ethnicity. If there was no information on smoking, then they were allocated the most frequent category for people of their age, gender, and ethnicity. Performance of QRISK3 in both Aurum and GOLD was assessed through discrimination and calibration. Discrimination was assessed using Harrell’s C-statistic which ranges between 0.5 (even chance) and 1 (perfect discrimination). Calibration was assessed in three ways: (i) ratio of expected and observed probability of CVD, (ii) calibration slope by estimating the beta-coefficient of the linear predictor of the score via a flexible parametric survival model for CVD, and (iii) a calibration plot of observed and expected probabilities for each tenth of predicted risk forming 10 equal sized groups.

#### Determination of risk factors and development of new model

Determination of key risk factors and new model development was performed in Aurum. Unadjusted and adjusted associations between risk factors and time to cardiovascular event were modelled using flexible parametric models with three degrees of freedom. Five models were considered overall, building in complexity. Model 1 included only demographic information (age centred around the mean; gender; ethnicity; and deprivation). Model 2 (reduced model) included risk factors the research team considered to be the key risk factors for cardiovascular disease: age, gender, ethnicity, deprivation, smoking status, type 1 diabetes, type 2 diabetes, family history of coronary event, chronic kidney disease, atrial fibrillation, treated hypertension, and body mass index. Model 3 included all covariates. Model 4 tested fractional polynomials for age and total cholesterol/HDL ratio and then used backwards stepwise selection (based on *P* < 0.01) of the factors in Model 3, with enforced entry of age, gender, and ethnicity. The full model (Model 5) assessed through backwards stepwise selection interactions of age and gender with the covariates remaining in Model 4.

#### Internal validation

For each model, the 10-year estimated risk of CVD was calculated for each patient. Discrimination was assessed using the C-statistic, and the D statistic where higher values indicate greater discrimination with an increase of ≥0.1 over other prediction models suggesting improved separation. Calibration was assessed as described for the assessment of QRISK3. The amount of optimism in the models was assessed using van Houwelingen’s heuristic shrinkage factor using 83 degrees of freedom.^23^

The net reclassification index (NRI) was derived comparing risk categorization on the QRISK3 to risk obtained from the optimal developed model using a risk of ≥10% as the cut-off as this is the level at which QRISK3 defines patients at high risk for CVD.

#### External validation

External validation of the five estimated risk equations from the above models was assessed in the CPRD GOLD data set. C-statistic, calibration slope, and ratio of expected and observed probability were determined. For the optimal models, discrimination and calibration by gender, geographical region (ten regions), and deprivation category (based on quintile scores) were also assessed.

#### Reduction in modifiable risk factors

The extent that risk of CVD could be reduced was determined by assessing potential impact of a population level reduction in two modifiable risk factors, using CPRD Aurum. Changes in risk factors considered were (i) move from current smoker to ex-smoker; (ii) move from obese to overweight; and (ii) move from obese to overweight or from current smoker to ex-smoker. The estimated reduction in mean 10-year risk was determined for each of these changes.

#### Sensitivity analyses

The first sensitivity analysis excluded patients prescribed lipid-lowering drugs during the diagnostic window. The second sensitivity analysis imputed missing data for smoking status, BMI, and cholesterol using multiple imputation by chained equations. All covariates plus the optimal fractional polynomials for age identified in Model 4, indicator for cardiovascular event, and time to cardiovascular event were included in the multiple imputation model. A two-stage procedure was conducted to identify the optimal number of imputed data sets required.^24^ Ten data sets were first imputed to determine the total number of imputed data sets required to ensure standard errors of hazard ratios could be replicated. In total, 20 imputed data sets were created for analysis. Finally, gender-specific models were developed and compared with the model incorporating gender-covariate interactions.

### Patient and public involvement

Three meetings with a patient and public users group were held. These meetings highlighted key risk factors from the patient perspective, discussed interpretations of findings, and potential use by patients of the information resulting from the study.

## Results

In the development data set (Aurum), 374 917 patients had a new record of unattributed chest pain, fulfilled the inclusion criteria, and had complete linkage. Mean age was 47.8 (SD 16.5) years and 47% were male. Median follow-up was 6.1 years. There were 226 024 patients in the validation data set (GOLD) with similar mean age (47.3) and percentage who were male (47%), but with a shorter median follow-up of 5.4 years. Baseline characteristics are shown in *Table 1* (development data set) and supplementary material online, *Table S2* (validation data set).

### QRISK3

In Aurum, although the C-statistic was high (0.79 overall, 0.79 males, 0.80 females), QRISK3 underestimated the risk of CVD, with the amount of underestimation becoming larger in higher-risk groups (*Table 2*, supplementary material online, *Figure S1*). The estimated calibration slope was 0.75 (overall), 0.83 (males) and 0.78 (females) and the ratio of expected and observed probability of CVD was 0.51 (overall), 0.53 (males) and 0.48 (females). Similar performance measures were observed in GOLD (*Table 3*, supplementary material online, *Figure S1*).

**Table 2:** Performance of QRISK 3 and internal validation for risk prediction models for unattributed chest pain in development data set

	QRISK3	Model 1	Model 2 (reduced)	Model 3	Model 4	Model 5 (full)
*All*						
C-statistic (95% CI)	0.789 (0.787, 0.791)	0.777 (0.775, 0.779)	0.793 (0.791, 0.795)	0.796 (0.794, 0.798)	0.796 (0.794, 0.798)	0.797 (0.795, 0.799)
D statistic (SE)	—	1.743 (0.009)	1.853 (0.009)	1.880 (0.009)	1.877 (0.009)	1.881 (0.009)
Calibration slope (95% CI)	0.752 (0.738, 0.765)	1 (0.985, 1.015)	1 (0.985, 1.015)	1 (0.985, 1.015)	1 (0.984, 1.016)	1 (0.984, 1.016)
E/O event probabilities at 10 yrs	0.505	1.004	1.007	1.009	1.007	1.008
Baseline survival at 10 yrs	—	0.8825521	0.9154936	0.9212328	0.9170177	0.9128141
von Houwelingen’s heuristic shrinkage factor (df = 83)	—	0.998	0.998	0.998	0.998	0.998
*Males*						
C-statistic (95% CI)	0.792 (0.789, 0.795)	0.776 (0.773, 0.779)	0.791 (0.788, 0.794)	0.793 (0.791, 0.796)	0.794 (0.791, 0.796)	0.793 (0.790, 0.796)
Calibration slope (95% CI)	0.826 (0.808, 0.844)	1.000 (0.983, 1.016)	0.993 (0.977, 1.009)	0.995 (0.979, 1.011)	0.990 (0.973, 1.006)	0.999 (0.982, 1.017)
E/O event probabilities at 10 yrs	0.530	1.002	1.006	1.008	1.006	1.009
*Females*						
C-statistic (95% CI)	0.797 (0.794, 0.800)	0.776 (0.773, 0.780)	0.794 (0.791, 0.797)	0.798 (0.795, 0.801)	0.798 (0.795, 0.801)	0.799 (0.796, 0.802)
Calibration slope (95% CI)	0.776 (0.757, 0.794)	1.000 (0.981, 1.020)	1.007 (0.988, 1.027)	1.005 (0.987, 1.024)	1.011 (0.990, 1.031)	1.001 (0.982, 1.020)
E/O event probabilities at 10 yrs	0.480	1.006	1.009	1.010	1.009	1.006

Model 1: Sociodemographic factors only; Model 2: Traditional risk factors; Model 3: All covariates; Model 4: Backwards selection and inclusion of fractional polynomials for age and total cholesterol level/HDL ratio; Model 5: Addition to Model 4 of interaction terms of gender with age, ethnic group, type 2 diabetes, atrial fibrillation, hypertension, BMI, and respiratory conditions.

E, expected; O, observed.

**Table 3:** Performance of QRISK3 and external validation of Models 2 and 5 in validation data set

	QRISK3	Model 2 (reduced)	Model 5 (full)
*All*			
C-statistic (95% CI)	0.798 (0.795, 0.801)	0.802 (0.799, 0.805)	0.805 (0.802, 0.807)
Calibration slope (95% CI)	0.767 (0.747, 0.786)	1.022 (1.000, 1.043)	1.022 (0.998, 1.046)
E/O event probabilities at 10 yrs	0.493	1.000	0.997
*Males*			
C-statistic (95% CI)	0.801 (0.797, 0.804)	0.799 (0.795, 0.803)	0.801 (0.797, 0.805)
Calibration slope (95% CI)	0.851 (0.826, 0.876)	1.002 (0.980, 1.024)	1.014 (0.990, 1.039)
E/O event probabilities at 10 yrs	0.517	0.995	0.998
*Females*			
C-statistic (95% CI)	0.805 (0.801, 0.809)	0.804 (0.800, 0.808)	0.806 (0.802, 0.810)
Calibration slope (95% CI)	0.789 (0.763, 0.814)	1.041 (1.013, 1.070)	1.029 (0.999, 1.059)
E/O event probabilities at 10 yrs	0.468	1.005	0.997

E, expected; O, observed

### Determination of risk factors and model development

The associations with CVD for each of the risk factors across Models 1–4 in the development data set are shown in *Table 4*. Most risk factors were shown to increase the risk of CVD in patients with unattributed chest pain. Backwards selection (model 4) only removed osteoarthritis, although chronic kidney disease had a moderate association and was not statistically significant in Models 2–3. The strongest comorbid risk factors included type 1 diabetes (model 4 adjusted HR 2.41; 95% CI 2.11, 2.76), atrial fibrillation (1.95; 1.85, 2.06), and treated hypertension (1.55; 1.50, 1.59). Sociodemographic risk factors included older age, male gender, living in more deprived areas, and Asian populations. Black populations and patients in other ethnic groups had lower risk (compared to White populations). Being a current smoker, obesity and being overweight also increased the risk of CVD.

**Table 4:** Adjusted hazard ratios (95% confidence interval) for cardiovascular disease in patients with unattributed chest pain (development data set)

	Model 1:	Model 2:	Model 3:	Model 4:
Age^a^	1.07 (1.07, 1.07)	1.06 (1.06, 1.06)	1.06 (1.06, 1.06)	
Age^b^				17.52 (15.77, 19.45)
Age^c^				1.00 (1.00, 1.00)
Ethnicity				
White/not recorded	1	1	1	1
Asian	1.13 (1.07, 1.20)	1.16 (1.10, 1.23)	1.16 (1.10, 1.23)	1.16 (1.09, 1.23)
Black	0.77 (0.72, 0.83)	0.74 (0.69, 0.79)	0.78 (0.73, 0.83)	0.78 (0.73, 0.83)
Other	0.82 (0.73, 0.91)	0.85 (0.76, 0.95)	0.87 (0.78, 0.97)	0.87 (0.78, 0.97)
Deprivation				
Least	1	1	1	1
2n^d^	1.09 (1.05, 1.13)	1.07 (1.03, 1.11)	1.06 (1.02, 1.10)	1.06 (1.02, 1.10)
3r^d^	1.19 (1.14, 1.23)	1.14 (1.10, 1.19)	1.13 (1.08, 1.17)	1.13 (1.09, 1.18)
4th	1.33 (1.27, 1.39)	1.23 (1.18, 1.28)	1.21 (1.15, 1.26)	1.22 (1.16, 1.27)
Most	1.53 (1.45, 1.61)	1.37 (1.30, 1.44)	1.33 (1.26, 1.40)	1.34 (1.27, 1.41)
Sex: females vs. males	0.70 (0.68, 0.71)	0.71 (0.69, 0.72)	0.71 (0.69, 0.72)	0.72 (0.70, 0.73)
Smoking status				
Never		1	1	1
Current		1.50 (1.45, 1.55)	1.45 (1.41, 1.50)	1.44 (1.40, 1.49)
Ex		1.14 (1.11, 1.17)	1.11 (1.08, 1.14)	1.10 (1.07, 1.13)
Not recorded		1.16 (1.08, 1.24)	1.17 (1.09, 1.25)	1.19 (1.12, 1.28)
Diabetes type 1		2.33 (2.03, 2.67)	2.42 (2.11, 2.77)	2.41 (2.11, 2.76)
Diabetes type 2		1.30 (1.25, 1.35)	1.31 (1.27, 1.36)	1.31 (1.26, 1.36)
FH angina/heart attack <60 yrs		1.23 (1.18, 1.29)	1.24 (1.18, 1.29)	1.20 (1.15, 1.26)
CKD diagnosis/eGFR < 60		1.02 (0.99, 1.06)	1.01 (0.97, 1.04)	1.04 (1.01, 1.08)
Atrial fibrillation		1.90 (1.80, 2.00)	1.90 (1.81, 2.01)	1.95 (1.85, 2.06)
Treated hypertension		1.55 (1.50, 1.59)	1.54 (1.49, 1.58)	1.55 (1.50, 1.59)
Migraine			1.11 (1.04, 1.19)	1.11 (1.04, 1.19)
Rheumatoid arthritis			1.28 (1.18, 1.39)	1.26 (1.16, 1.37)
Severe mental illness			1.27 (1.19, 1.36)	1.26 (1.18, 1.35)
Corticosteroids			1.30 (1.26, 1.34)	1.30 (1.26, 1.35)
Body mass index				
Underweight/normal		1	1	1
Overweight		1.06 (1.03, 1.09)	1.04 (1.01, 1.07)	1.02 (0.99, 1.04)
Obese		1.29 (1.25, 1.33)	1.23 (1.20, 1.27)	1.19 (1.15, 1.22)
Not recorded		1.07 (1.03, 1.12)	1.08 (1.03, 1.13)	1.09 (1.04, 1.13)
Depression/anxiety			1.14 (1.11, 1.17)	1.13 (1.10, 1.17)
Oesophageal reflux			1.06 (1.03, 1.10)	1.06 (1.03, 1.09)
Respiratory			1.17 (1.14, 1.20)	1.18 (1.15, 1.21)
Osteoarthritis			1.02 (0.98, 1.05)	
Low back pain			1.07 (1.04, 1.10)	1.07 (1.04, 1.10)
Neck pain			1.06 (1.02, 1.10)	1.05 (1.02, 1.09)
Cancer			1.12 (1.06, 1.17)	1.13 (1.08, 1.19)
Cholesterol/HDL ratio^d^			1.07 (1.06, 1.08)	
Cholesterol/HDL ratio^e^				4.59 (3.62, 5.80)
Cholesterol/HDL ratio^f^				0.51 (0.42, 0.62)

Model 1: Sociodemographic factors only; Model 2: Traditional risk factors; Model 3: All covariates; Model 4: Backwards selection and fractional polynomials for age and total cholesterol level/HDL ratio.

HDL, high-density lipoprotein.

Age and total cholesterol level/HDL ratio are transformed as follows:

age—47.76824

ln(age/10)—1.563775822

(age/10)^3—108.9977745

(total cholesterol/HDL ratio)—3.909598

(total cholesterol level/HDL ratio/10)^2—0.1528495691

(total cholesterol level/HDL ratio/10)^3—0.0597580377

Interactions of gender with age, ethnicity, type 2 diabetes, atrial fibrillation, treated hypertension, respiratory conditions, and BMI were included in Model 5, suggesting that their impact varies by gender with slightly higher increased risk if a comorbidity is present in females than males, but lower risk related to obesity and in Asian populations. No interactions were observed for age other than with gender. This matched findings from the sensitivity analysis developing gender-specific models which showed consistency in risk factors between males and females, although generally with slightly stronger associations in females for comorbidity (see supplementary material online, *Table S3*).

Sensitivity analysis removing patients prescribed lipid-lowering drugs and imputation of missing data yielded similar hazard ratios as the main analysis (data not shown).

### Internal validation

Internal validation showed predictive performance was good across the five models and better than QRISK3 (*Table 2*). C-statistic values ranged between 0.78 and 0.80. The D statistic values suggest Models 2–5 had greater discriminative ability than Model 1 although there was little difference in discrimination ability between these four models. There was close agreement between the observed and predictive probabilities for CVD. There was a negligible amount of optimism so estimated coefficients were not corrected for this. Performance was good when stratified by gender (*Table 2*).

**Figure 1:**
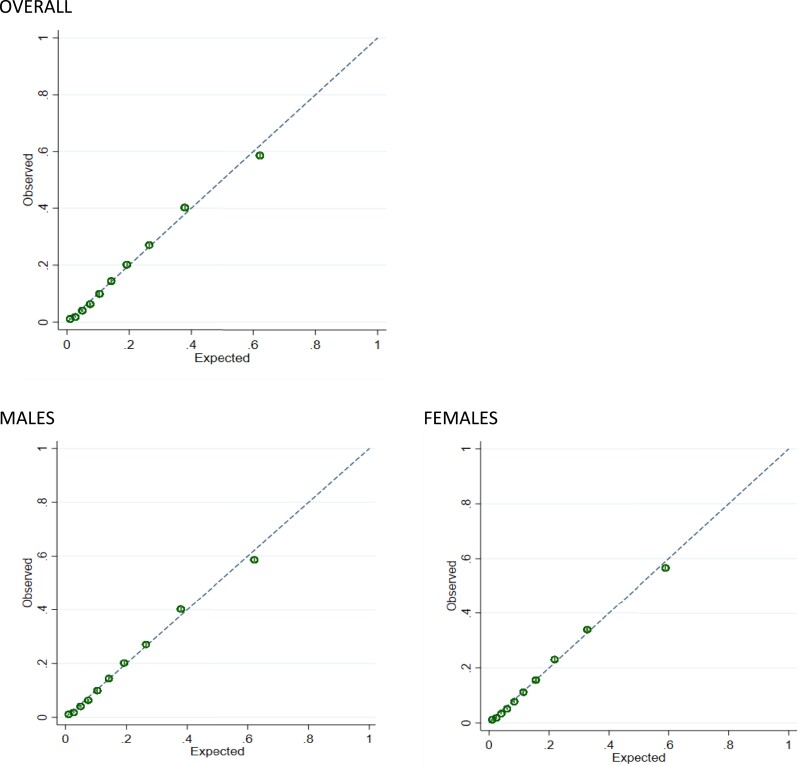
Internal validation overall and by gender: calibration plots for full model (Model 5) in development data set.

Calibration plots showed good agreement between observed and predicted CVD at all levels of risk overall, and by gender (plots for Model 5 shown in *Figure 1*).

### Comparison to QRISK3

Model 5 estimated 53% of patients had a 10-year CVD risk of 10% or more, compared to 29% using QRISK3. Only 188 (0.1%) patients with risk more than 10% on QRISK3 moved to a risk estimate of less than 10% on the new model. Net reclassification index for events (net proportion of patients with events assigned a higher risk category based on ≥10% cut-off) was 0.21 and for non-events (net proportion of patients without events assigned a lower risk category) was −0.24.

### External validation

**Figure 2:**
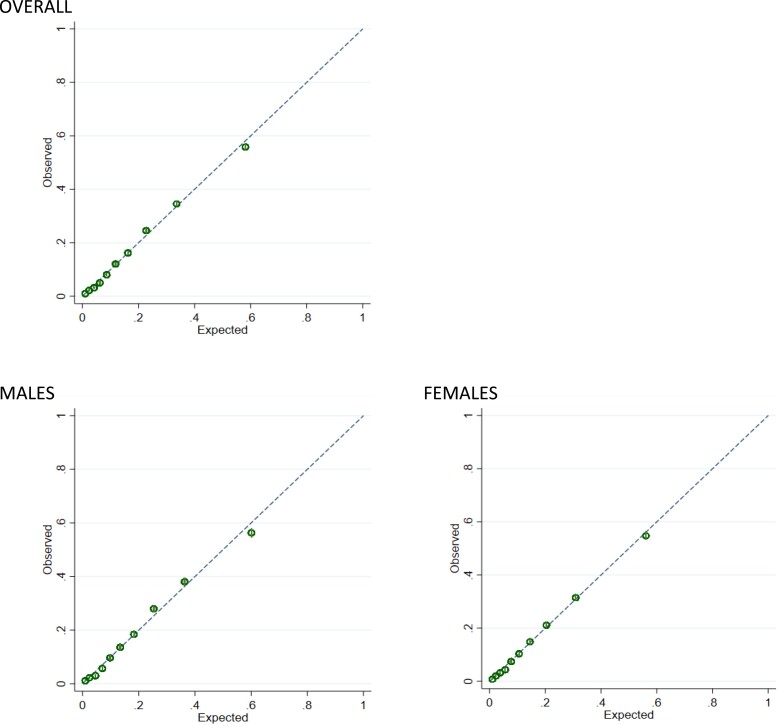
External validation overall and by gender: calibration plots for full model (Model 5) in validation data set.

The models showed strong predictive performance in the external validation data set, overall and by gender, and were again superior to QRISK3 (*Table 3*). Model 5 C-statistic was 0.81 and the ratio of expected and observed probabilities for CVD and calibration slopes were close to one. Calibration plots for Model 5 (*Figure 2*) show good agreement between observed and expected risk at all levels of risk. Stratified by deprivation, Model 5 C-statistics ranged from 0.80 to 0.81 and calibration slopes from 0.91 to 1.09 and stratified by geographical region, C-statistics ranged from 0.79 to 0.82 and calibration slopes from 0.94 to 1.08 (see supplementary material online, *Figures S2* and *S3*). Model 5 also performed well in those currently not prescribed lipid-lowering drugs (C-statistic 0.81, calibration slope 1.05) and performed as well as gender-specific models in terms of discrimination and calibration (see supplementary material online, *Table S4*). The reduced Model 2 based on traditional risk factors also gave good model performance that was similar to the full Model 5 in the validation data set (*Table 3*).

The risk equations based on Models 2 and 5 are shown in supplementary material online, *Table S5*.

### Modifiable risk factors

Nearly half of patients with a Model 5 estimated CVD risk of ≥10% were either current smokers or obese. Population level removal of these factors reduced the estimated mean 10-year risk from 17.4% to 16.9% (all obese to overweight), 16.5% (all current to ex-smoker), and 16.0% (all obese to overweight and all current to ex-smoker). The biggest estimated effect is in those who are obese and currently smoke where removal of both factors would reduce estimated risk from 21.7% to 15.6%.

## Discussion

This study of over 600 000 patients with unattributed chest pain has identified their key risk factors for future cardiovascular disease recorded in primary care, highlighted that general population algorithms will underestimate CVD risk in this population, derived improved prediction models with high discrimination and calibration, and validated these findings in a second database.

There are several cardiovascular risk prediction algorithms for potential use for clinicians.^25,26^ UK primary care guidelines recommend the use of QRISK for prediction of 10-year cardiovascular risk.^10^ However, this algorithm was designed and validated for use in the general population, not in those presenting with chest pain who are older, have increased risk of a future cardiovascular event, and may already be prescribed lipid-lowering medication.^9^ It is not surprising therefore that QRISK3 underestimates the cardiovascular risk in this population. A third of patients classified below the recommended 10% cut-off for starting preventative medication on QRISK3 were classified as having risk greater than 10% based on our developed model. By contrast, only 0.1% of patients with risk greater than 10% on QRISK3 had risk below that level on the developed model. However, other than a less strong association of chronic kidney disease, there was consistency in the key risk factors identified in this population with those identified by QRISK3 for the UK general population. This includes the higher risk associated with Asian populations and lower risk for Black populations. Whilst there are conflicting findings from other studies relating to risk for Black populations, a reduced risk has also been identified in other UK general population studies^27^ and other studies have shown an increased risk of cardiovascular disease in Black patient groups is removed after adjustment for other risk factors such as socioeconomic characteristics.^28,29^ Our study indicates risk of cardiovascular disease is higher in males, which has also been shown in patients discharged from hospital with unexplained chest pain.^30^ Despite indications in our study from our full model (Model 5) that certain comorbidities (type 2 diabetes, atrial fibrillation, hypertension, and respiratory conditions) confer higher risk in females than males, and being obese a lower risk, the full prediction model including interaction terms with gender did not greatly improve the model compared to models without interaction, and gender-specific models also did not improve performance. Higher risk related to some comorbidity for females seen in the unattributed chest pain population is also generally evident for the general population.^10,31^

### Implications

There is a high proportion of patients presenting with chest pain that, whilst not typical of ischaemic chest pain, cannot be attributed definitively to another cause, and these patients are at higher risk of future CVD than those with chest pain attributed to a non-coronary cause or without chest pain.^4,7–9^ A survey of UK GPs in 2019 found that most respondents are aware of cardiovascular risk prediction tools and QRISK in particular, and use them to guide therapy and to comply with guidelines.^32^ However, studies have also shown that preventative medication is not always targeted at those most at risk.^33–35^ This includes those patients presenting with chest pain with an unattributed cause^9^ and that will be magnified as our current analysis shows that the risk of future CVD is likely to be underestimated by CVD risk prediction tools recommended for use in primary care as a method of ascertaining who should receive preventative measures. Whilst the most optimal model developed in those with unattributed chest pain includes a range of covariates and interactions, a simpler model utilizing just traditional risk factors and without interactions can be used without great loss of performance (as found in other populations^36^). A small number of key risk factors can hence be used to accurately predict CVD, and it likely that GPs should focus on these factors as a means of targeting for closer surveillance and management. This could include encouragement and initiatives to improve lifestyle behaviours relating to diet, physical activity, and smoking, and prescribing of lipid-lowering and other preventative medication. This study has also shown the potential benefit of lifestyle behaviour relating to smoking and diet, with a potential reduction for those currently obese and who smoke from 22% to 16% in mean 10-year CVD risk.

### Strengths and limitations

This study utilized two large, nationally representative primary care EHR databases,^12,14^ representing different information systems used in UK primary care, with linkage to inpatient, mortality, and deprivation data. The list of potential risk factors was wide and drew on those used in other UK cardiovascular prediction algorithms. Models performed well across genders, deprivation levels, and geographical regions.

A coded primary care record of chest pain with no attribution does not indicate any suspected underlying reason for the chest pain the primary care provider may have. This may be recorded in free (unstructured) text that generally cannot be accessed for research. The coded record though should reflect findings from any cardiac diagnostic investigation. We excluded patients with recorded cardiovascular events in the first 6 months as they were likely to be the underlying reason for the initial presentation of chest pain. It is possible the diagnostic period may be longer than 6 months for some patients prior to a cardiovascular event being diagnosed, particularly if the patient presented with atypical features. However, rapid access chest pain clinics in the UK should ensure that most patients receive a diagnosis within 6 months. Some patients were already being prescribed lipid-lowering drugs and the algorithm may be best used on those not currently offered such preventative medication. However, the magnitude and direction of the risk factor estimates were similar in the subgroup of those not prescribed lipid-lowering medication to those for the overall models. As is common in routine primary care data, there was missing data on smoking, BMI, and cholesterol. A sensitivity analysis using multiple imputation suggested the missing data would not impact on findings. There was a low percentage of patients recorded as non-white in the validation data set. Further research should test the models in different ethnic groups.

## Conclusions

Patients presenting to primary care with unattributed chest pain are at increased risk of cardiovascular events, but this study has shown that it is feasible to ascertain those most at risk using routinely recorded information in the primary care record. Consideration of a select number of key risk factors identified here could help target patients at highest risk for preventative measures.

## Author contributions

K.P.J., T.R.-M., D.A.v.d.W., and M.A.M. designed the study. K.P.J. and T.R.-M. wrote the analysis plan and T.R.-M. performed the analysis. J.B. and K.P.J. prepared the data. K.P.J., S.D., R.A.H., and M.A.M. defined code lists for exposure, covariates, and outcomes. All authors interpreted the findings. K.P.J. and T.R.-M. drafted the paper. All authors contributed to revision of the paper and have approved the final version.

Kelvin Jordan, Trishna Rathod-Mistry, Danielle A van der Windt, James Bailey, Ying Chen, Lorna Clarson, Spiros Denaxas, Richard A Hayward, Harry Hemingway, Theocharis Kyriacou, and Mamas A Mamas

### Ethics

The study was approved by the CPRD Independent Scientific Advisory Committee, ref 19_205. The approved protocol was made available to reviewers.

## Supplementary material

Supplementary material is available at *European Journal of Preventive Cardiology* online.

## Supplementary Material

zwad055_Supplementary_Data

## Data Availability

Data may be obtained from a third party and are not publicly available. The data were obtained from the Clinical Practice Research Datalink (CPRD). CPRD data governance does not allow us to distribute patient data to other parties. Researchers may apply for data access at http://www.CPRD.com/.

## References

[1] Ruigómez A , Rodríguez LAG, Wallander M-A, Johansson S, Jones R. Chest pain in general practice: incidence, comorbidity and mortality. Fam Pract 2006;23:167–174. 16461444 10.1093/fampra/cmi124

[2] Walters K , Rait G, Hardoon S, Kalaitzaki E, Petersen I, Nazareth I. Socio-demographic variation in chest pain incidence and subsequent coronary heart disease in primary care in the United Kingdom. Eur J Prev Cardiol 2014;21:566–575. 22617118 10.1177/2047487312449415

[3] Ruigómez A , Massó-González EL, Johansson S, Wallander MA, García-Rodríguez LA. Chest pain without established ischaemic heart disease in primary care patients: associated comorbidities and mortality. Br J Gen Pract 2009;59:e78–e86. 19275827 10.3399/bjgp09X407054 PMC2648936

[4] Jordan KP , Timmis A, Croft P, van der Windt DA, Denaxas S, González-Izquierdo A, et al. Prognosis of undiagnosed chest pain: linked electronic health record cohort study. BMJ 2017;357:j1194. 28373173 10.1136/bmj.j1194 PMC5482346

[5] Haasenritter J , Biroga T, Keunecke C, Becker A, Donner-Banzhoff N, Dornieden K, et al Causes of chest pain in primary care—a systematic review and meta-analysis. Croat Med J 2015;56:422–430. 26526879 10.3325/cmj.2015.56.422 PMC4655927

[6] Robson J , Ayerbe L, Mathur R, Addo J, Wragg A. Clinical value of chest pain presentation and prodromes on the assessment of cardiovascular disease: a cohort study. BMJ Open 2015;5:e007251. 10.1136/bmjopen-2014-007251 PMC4401860 25877275

[7] Croft PR , Thomas E. Chest pain and subsequent consultation for coronary heart disease: a prospective cohort study. Br J Gen Pract 2007;57:40–44. 17244423 PMC2032699

[8] Sekhri N , Feder GS, Junghans C, Hemingway H, Timmis AD. How effective are rapid access chest pain clinics? Prognosis of incident angina and non-cardiac chest pain in 8762 consecutive patients. Heart 2007;93:458–463. 16790531 10.1136/hrt.2006.090894 PMC1861500

[9] Jordan KP , Rathod-Mistry T, Bailey J, Chen Y, Clarson L, Denaxas S, et al Long-term cardiovascular risk and management of patients recorded in primary care with unattributed chest pain: an electronic health record study. J Am Heart Assoc 2022;11:e023146. 35301875 10.1161/JAHA.121.023146 PMC9075433

[10] Hippisley-Cox J , Coupland C, Brindle P. Development and validation of QRISK3 risk prediction algorithms to estimate future risk of cardiovascular disease: prospective cohort study. BMJ 2017;357:j2099. 28536104 10.1136/bmj.j2099 PMC5441081

[11] National Institute for Health and Care Excellence . Cardiovascular disease: risk assessment and reduction, including lipid modification, Clinical guideline [CG181]. July 2014. Available from https://www.nice.org.uk/guidance/cg181. 36952507

[12] Wolf A , Dedman D, Campbell J, Booth H, Lunn D, Chapman J, et al Data resource profile: Clinical Practice Research Datalink (CPRD) Aurum. Int J Epidemiol 2019;48:1740–1740g. 30859197 10.1093/ije/dyz034 PMC6929522

[13] Clinical Practice Research Datalink . https://www.cprd.com. Accessed 26 March 2021.

[14] Herrett E , Gallagher AM, Bhaskaran K, Forbes H, Mathur R, van Staa T, et al Data resource profile: Clinical Practice Research Datalink (CPRD). Int J Epidemiol 2015;44:827–836. 26050254 10.1093/ije/dyv098 PMC4521131

[15] Padmanabhan S , Carty L, Cameron E, Ghosh RE, Williams R, Strongman H. Approach to record linkage of primary care data from Clinical Practice Research Datalink to other health-related patient data: overview and implications. Eur J Epidemiology 2019;34:91–99. 10.1007/s10654-018-0442-4 PMC6325980 30219957

[16] Riley RD , Hayden JA, Steyerberg EW, Moons KG, Abrams K, Kyzas PA, et al Prognosis Research Strategy (PROGRESS) 2: prognostic factor research. PLoS Med 2013;10:e1001380. 23393429 10.1371/journal.pmed.1001380 PMC3564757

[17] Steyerberg EW , Moons KG, van der Windt DA, Hayden JA, Perel P, Schroter S, et al Prognosis Research Strategy (PROGRESS) 3: prognostic model research. PLoS Med 2013;10:e1001381. 23393430 10.1371/journal.pmed.1001381 PMC3564751

[18] Collins GS , Reitsma JB, Altman DG, Moons KG. Transparent reporting of a multivariable prediction model for individual prognosis or diagnosis (TRIPOD): the TRIPOD statement. BMJ 2015;350:g7594. 25569120 10.1136/bmj.g7594

[19] Denaxas SC , George J, Herrett E, et al Data resource profile: cardiovascular disease research using linked bespoke studies and electronic health records (CALIBER). Int J Epidemiol 2012;41:1625–1638. 23220717 10.1093/ije/dys188 PMC3535749

[20] Rapsomaniki E , Shah A, Perel P, Denaxas S, George J, Nicholas O, et al Prognostic models for stable coronary artery disease based on electronic health record cohort of 102 023 patients. Eur Heart J 2014;35:844–852. 24353280 10.1093/eurheartj/eht533 PMC3971383

[21] Pasea L , Chung SC, Pujades-Rodriguez M, Moayyeri A, Denaxas S, Fox KAA, et al Personalising the decision for prolonged dual antiplatelet therapy: development, validation and potential impact of prognostic models for cardiovascular events and bleeding in myocardial infarction survivors. Eur Heart J 2017;38:1048–1055. 28329300 10.1093/eurheartj/ehw683 PMC5400049

[22] ClinRisk Ltd . QRISK®3-2017 risk calculator. 1 (2017). Available at https://qrisk.org/three/index.php. Accessed: 26 March 2021.

[23] van Houwelingen JC , Le Cessie S. Predictive value of statistical models. Stat Med 1990;9:1303–1325. 2277880 10.1002/sim.4780091109

[24] von Hippel PT . How many imputations do you need? A two-stage calculation using a quadratic rule. Sociol Methods Res 2020;49:699–718. 10.1177/0049124117747303 PMC11361408 39211325

[25] de Vries TI , Visseren FLJ. Cardiovascular risk prediction tools made relevant for GPs and patients. Heart 2021;107:332–340. 10.1136/heartjnl-2019-316377 33077500

[26] Ban JW , Wallace E, Stevens R, Perera R. Why do authors derive new cardiovascular clinical prediction rules in the presence of existing rules? A mixed methods study. PLoS One 2017;12:e0179102. 28591223 10.1371/journal.pone.0179102 PMC5462434

[27] George J , Mathur R, Shah AD, Pujades-Rodriguez M, Denaxas S, Smeeth L, et al Ethnicity and the first diagnosis of a wide range of cardiovascular diseases: associations in a linked electronic health record cohort of 1 million patients. PLoS One 2017;12:e0178945. 28598987 10.1371/journal.pone.0178945 PMC5466321

[28] Shah NS , Ning H, Petito LC, Kershaw KN, Bancks MP, Reis JP, et al Associations of clinical and social risk factors with racial differences in premature cardiovascular disease. Circulation 2022;146:201–210. 35607988 10.1161/CIRCULATIONAHA.121.058311 PMC9308688

[29] Ho FK , Gray SR, Welsh P, Gill JMR, Sattar N, Pell JP, et al Ethnic differences in cardiovascular risk: examining differential exposure and susceptibility to risk factors. BMC Med 2022;20:149. 35473626 10.1186/s12916-022-02337-w PMC9042646

[30] Egeland GM , Akerkar R, Kvåle R, Sulo G, Tell GS, Bakken IJ, et al Hospitalised patients with unexplained chest pain: incidence and prognosis. J Intern Med 2019;286:562–572. 31322304 10.1111/joim.12948

[31] Mehta S , Jackson R, Pylypchuk R, Poppe K, Wells S, Kerr AJ. Development and validation of alternative cardiovascular risk prediction equations for population health planning: a routine health data linkage study of 1.7 million New Zealanders. Int J Epidemiol 2018;47:1571–1584. 30010781 10.1093/ije/dyy137

[32] Ban JW , Perera R, Stevens R. GPs’ familiarity with and use of cardiovascular clinical prediction rules: a UK survey study. BJGP Open 2020;4:bjgpopen20X101081. 10.3399/bjgpopen20X101081 PMC7880194 33023870

[33] Wu J , Zhu S, Yao GL, Mohammed MA, Marshall T. Patient factors influencing the prescribing of lipid lowering drugs for primary prevention of cardiovascular disease in UK general practice: a national retrospective cohort study. PLoS ONE 2013;8:e67611. 23922649 10.1371/journal.pone.0067611 PMC3724846

[34] van Staa T-P , Smeeth L, Ng ES-W, Goldacre B, Gulliford M. The efficiency of cardiovascular risk assessment: do the right patients get statin treatment? Heart 2013;99:1597–1602. 23735939 10.1136/heartjnl-2013-303698 PMC3812879

[35] Finnikin S , Ryan R, Marshall T. Statin initiations and QRISK2 scoring in UK general practice: a THIN database study. Br J Gen Pract 2017;67:e881–e887. 29061715 10.3399/bjgp17X693485 PMC5697558

[36] Pylypchuk R , Wells S, Kerr A, Poppe K, Riddell T, Harwood M, et al Cardiovascular disease risk prediction equations in 400 000 primary care patients in New Zealand: a derivation and validation study. Lancet 2018;391:1897–1907. 29735391 10.1016/S0140-6736(18)30664-0

