# Supplementary material for: Determining cardiovascular risk in patients with unattributed chest pain in UK primary care: an electronic health record study
Source: Eur J Prev Cardiol. 2023 Mar 10;30(11):1151–61. doi: 10.1093/eurjpc/zwad055 (PMC10442054; doi:10.1093/eurjpc/zwad055)
Supplement: zwad055_Supplementary_Data [file zwad055_supplementary_data.docx]

Supplementary table 1 – Code lists for chest pain and cardiovascular disease

| **UNATTRIBUTED CHEST PAIN** |  |
| --- | --- |
| **Term** | **Read code** |
| Chest pain | 182 |
| Central chest pain | 1822 |
| Precordial pain | 1823 |
| Anterior chest wall pain | 1824 |
| Parasternal pain | 1826 |
| Atypical chest pain | 1828 |
| Retrosternal pain | 1829 |
| Chest pain on exertion | 182A |
| Costal margin chest pain | 182B0 |
| Chest wall pain | 182C |
| Chest pain NOS | 182Z |
| Seen in rapid access chest pain clinic | 9N0f |
| [D]Chest pain | R065 |
| [D]Precordial pain | R0651 |
| [D]Anterior chest wall pain | R0652 |
| [D]Chest discomfort | R0656 |
| [D]Parasternal chest pain | R0659 |
| [D]Chest pain NOS | R065z |
| [D]Chest pain, unspecified | R0650 |
| [D]Retrosternal chest pain | R065C |
| [D]Central chest pain | R065D |
| [D] Retrosternal chest pain | R0650-1 |
| [D]Chest pressure | R0657 |
| [D]Chest tightness | R0658 |
| [X]Other chest pain | Ryu04 |
|  |  |
| **Term** | **ICD 10 code** |
| Precordial pain | R07.2 |
| Other chest pain | R07.3 |
| Anterior chest-wall pain NOS | R07.3 |
| Chest pain, unspecified | R07.4 |
|  |  |
| **CARDIOVASCULAR DISEASE** |  |
| **Term** | **Read code** |
| H/O: angina pectoris | 14A5 |
| H/O: Angina in last year | 14AJ |
| Frequency of angina | 187 |
| Angina self-management plan agreed | 661M0 |
| Angina self-management plan review | 661N0 |
| Angina control | 662K |
| Angina control - good | 662K0 |
| Angina control - poor | 662K1 |
| Angina control - improving | 662K2 |
| Angina control - worsening | 662K3 |
| Angina self management plan commenced | 662K4 |
| Angina self management plan completed | 662K5 |
| Angina control NOS | 662Kz |
| Antianginal therapy | 8B27 |
| Referral to Angina Plan self-management programme declined | 8IEY |
| Referral to Angina Plan self-management programme | 8T04 |
| Crescendo angina | G311-1 |
| Impending infarction | G311-2 |
| Unstable angina | G311-3 |
| Angina at rest | G311-4 |
| Unstable angina | G3111 |
| Angina at rest | G3112 |
| Refractory angina | G3113 |
| Worsening angina | G3114 |
| Acute coronary insufficiency | G31y0 |
| Angina pectoris | G33 |
| Angina decubitus | G330 |
| Nocturnal angina | G3300 |
| Angina decubitus NOS | G330z |
| Prinzmetal's angina | G331 |
| Variant angina pectoris | G331-1 |
| Coronary artery spasm | G332 |
| Angina pectoris NOS | G33z |
| Status anginosus | G33z0 |
| Syncope anginosa | G33z2 |
| Angina on effort | G33z3 |
| Ischaemic chest pain | G33z4 |
| New onset angina | G33z6 |
| Stable angina | G33z7 |
| Angina pectoris NOS | G33zz |
| Chronic coronary insufficiency | G34y0 |
| Cardiac syndrome X | G37 |
| [X]Other forms of angina pectoris | Gyu30 |
| [RFC] Angina | HNG0012 |
| Angina control - stable | EMISAC1 |
| Angina control - unsatisfactory | EMISAC3 |
| Angina grading (canadian cardiovascular society) | EMISAN1 |
| Anginal Control: No attacks | EMISHGT65 |
| Anginal pain | EMISCAN2 |
| Cause of Death- Angina Pectoris | EGTON2G44 |
| Unstable angina | EGTON458 |
| Coronary artery bypass graft operations | 792-1 |
| Saphenous vein graft replacement of coronary artery | 7920 |
| Saphenous vein graft bypass of coronary artery | 7920-1 |
| Saphenous vein graft replacement of one coronary artery | 79200 |
| Saphenous vein graft replacement of two coronary arteries | 79201 |
| Saphenous vein graft replacement of three coronary arteries | 79202 |
| Saphenous vein graft replacement of four+ coronary arteries | 79203 |
| Saphenous vein graft replacement of coronary artery OS | 7920y |
| Saphenous vein graft replacement coronary artery NOS | 7920z |
| Other autograft replacement of coronary artery | 7921 |
| Other autograft bypass of coronary artery | 7921-1 |
| Autograft replacement of one coronary artery NEC | 79210 |
| Autograft replacement of two coronary arteries NEC | 79211 |
| Autograft replacement of three coronary arteries NEC | 79212 |
| Autograft replacement of four of more coronary arteries NEC | 79213 |
| Other autograft replacement of coronary artery OS | 7921y |
| Other autograft replacement of coronary artery NOS | 7921z |
| Allograft replacement of coronary artery | 7922 |
| Allograft bypass of coronary artery | 7922-1 |
| Allograft replacement of one coronary artery | 79220 |
| Allograft replacement of two coronary arteries | 79221 |
| Allograft replacement of three coronary arteries | 79222 |
| Allograft replacement of four or more coronary arteries | 79223 |
| Other specified allograft replacement of coronary artery | 7922y |
| Allograft replacement of coronary artery NOS | 7922z |
| Prosthetic replacement of coronary artery | 7923 |
| Prosthetic bypass of coronary artery | 7923-1 |
| Prosthetic replacement of one coronary artery | 79230 |
| Prosthetic replacement of two coronary arteries | 79231 |
| Prosthetic replacement of three coronary arteries | 79232 |
| Prosthetic replacement of four or more coronary arteries | 79233 |
| Prosthetic replacement of coronary artery NOS | 7923z |
| Revision of bypass for coronary artery | 7924 |
| Revision of bypass for one coronary artery | 79240 |
| Revision of bypass for two coronary arteries | 79241 |
| Revision of bypass for three coronary arteries | 79242 |
| Revision of bypass for four or more coronary arteries | 79243 |
| Other specified revision of bypass for coronary artery | 7924y |
| Revision of bypass for coronary artery NOS | 7924z |
| Connection of mammary artery to coronary artery | 7925 |
| Creation of bypass from mammary artery to coronary artery | 7925-1 |
| Double anastomosis of mammary arteries to coronary arteries | 79250 |
| LIMA sequential anastomosis | 79250-1 |
| RIMA sequential anastomosis | 79250-2 |
| Double implant of mammary arteries into coronary arteries | 79251 |
| Single anast mammary art to left ant descend coronary art | 79252 |
| Single anastomosis of mammary artery to coronary artery NEC | 79253 |
| LIMA single anastomosis | 79253-1 |
| RIMA single anastomosis | 79253-2 |
| Single implantation of mammary artery into coronary artery | 79254 |
| Connection of mammary artery to coronary artery OS | 7925y |
| Connection of mammary artery to coronary artery NOS | 7925z |
| Connection of other thoracic artery to coronary artery | 7926 |
| Double anastom thoracic arteries to coronary arteries NEC | 79260 |
| Single anastomosis of thoracic artery to coronary artery NEC | 79262 |
| Single implantation thoracic artery into coronary artery NEC | 79263 |
| Connection of other thoracic artery to coronary artery NOS | 7926z |
| Open angioplasty of coronary artery | 79275 |
| Other replacement of coronary artery | 792C |
| Replacement of coronary arteries using multiple methods | 792C0 |
| Other specified replacement of coronary artery | 792Cy |
| Replacement of coronary artery NOS | 792Cz |
| Other bypass of coronary artery | 792D |
| Other specified other bypass of coronary artery | 792Dy |
| Other bypass of coronary artery NOS | 792Dz |
| Mechanical complication of coronary bypass | SP003 |
| Coronary artery bypass graft occlusion | SP076 |
| [V]Presence of aortocoronary bypass graft | ZV457 |
| [V]Presence of coronary artery bypass graft | ZV45K |
| [V]Presence of coronary artery bypass graft - CABG | ZV45K-1 |
| H/O: cardiovascular disease | 14A |
| H/O: heart disease NOS | 14AA |
| H/O: Treatment for ischaemic heart disease | 14AL |
| Cardiac disease monitoring | 662 |
| Heart disease monitoring | 662-1 |
| CHD monitoring | 662N |
| Cardiac event recording | 662Y |
| Cardiac disease monitoring NOS | 662Z |
| Cardiovascular disease monitoring | 66f |
| Cardiovascular disease annual review | 66f0 |
| Cardiovascular disease interim monitoring | 66f1 |
| Coronary heart disease annual review | 6A2 |
| Coronary heart disease review | 6A4 |
| Repair of aneurysm of coronary artery | 79271 |
| Cardiac emergency monitoring | 8A51 |
| Coronary heart disease medication review | 8B3k |
| Admit ischaemic heart disease emergency | 8H2V |
| Coronary heart disease monitoring refused | 8I37 |
| Exception reporting: CHD quality indicators | 9h0 |
| Excepted from CHD quality indicators: Patient unsuitable | 9h01 |
| Excepted from CHD quality indicators: Informed dissent | 9h02 |
| Coronary heart disease monitoring administration | 9Ob |
| Attends coronary heart disease monitoring | 9Ob0 |
| Refuses coronary heart disease monitoring | 9Ob1 |
| Coronary heart disease monitoring default | 9Ob2 |
| Coronary heart disease monitoring 1st letter | 9Ob3 |
| Coronary heart disease monitoring 2nd letter | 9Ob4 |
| Coronary heart disease monitoring 3rd letter | 9Ob5 |
| Coronary heart disease monitoring verbal invitation | 9Ob6 |
| Coronary heart disease monitoring deleted | 9Ob7 |
| Coronary heart disease monitoring check done | 9Ob8 |
| Coronary heart disease monitoring telephone invite | 9Ob9 |
| Ischaemic heart disease | G3 |
| Arteriosclerotic heart disease | G3-1 |
| Atherosclerotic heart disease | G3-2 |
| IHD - Ischaemic heart disease | G3-3 |
| Other acute and subacute ischaemic heart disease | G31 |
| Myocardial infarction aborted | G3110 |
| MI - Myocardial infarction aborted | G3110-1 |
| Other acute and subacute ischaemic heart disease | G31y |
| Subendocardial ischaemia | G31y2 |
| Transient myocardial ischaemia | G31y3 |
| Other acute and subacute ischaemic heart disease NOS | G31yz |
| Other chronic ischaemic heart disease | G34 |
| Coronary atherosclerosis | G340 |
| Coronary artery disease | G340-2 |
| Ventricular cardiac aneurysm | G3410 |
| Other cardiac wall aneurysm | G3411 |
| Mural cardiac aneurysm | G3411-1 |
| Aneurysm of coronary vessels | G3412 |
| Atherosclerotic cardiovascular disease | G342 |
| Ischaemic cardiomyopathy | G343 |
| Silent myocardial ischaemia | G344 |
| Other specified chronic ischaemic heart disease | G34y |
| Chronic myocardial ischaemia | G34y1 |
| Other specified chronic ischaemic heart disease NOS | G34yz |
| Other chronic ischaemic heart disease NOS | G34z |
| Asymptomatic coronary heart disease | G34z0 |
| Other specified ischaemic heart disease | G3y |
| Ischaemic heart disease NOS | G3z |
| Other forms of heart disease | G5 |
| Other specified heart disease | G5y |
| Other ill-defined heart disease | G5yy |
| Other ill-defined heart disease NOS | G5yyz |
| Other heart disease NOS | G5yz |
| Heart disease NOS | G5z |
| [X]Ischaemic heart diseases | Gyu3 |
| [X]Other forms of acute ischaemic heart disease | Gyu32 |
| [X]Other forms of chronic ischaemic heart disease | Gyu33 |
| [X]Other forms of heart disease | Gyu5 |
| [RFC] Chronic heart disease (CHD) | HNG0010 |
| [RFC] Coronary heart disease | HNG0601 |
| Coronary heart disease care plan | EMISNQCO115 |
| Coronary heart disease confirmed | EMISNQCO148 |
| Coronary heart disease monitoring in primary care | EMISNQCO168 |
| Coronary heart disease monitoring in secondary care | EMISNQCO169 |
| H/O: coronary heart disease | EMISNOQFH3 |
| On coronary heart disease register | EMISNQON8 |
| Reason for influenza vaccine - chronic heart disease | EMISNQRE330 |
| CHD annual review | EMISQCH1 |
| H/O: heart failure | 14A6 |
| H/O: Heart failure in last year | 14AM |
| Paroxysmal nocturnal dyspnoea | 1736 |
| Suspected heart failure | 1J60 |
| Heart failure confirmed | 1O1 |
| O/E - pulmonary oedema | 23E1 |
| New York Heart Assoc classification heart failure symptoms | 388D |
| Echocardiogram shows left ventricular systolic dysfunction | 585f |
| Echocardiogram shows left ventricular diastolic dysfunction | 585g |
| Heart failure self-management plan agreed | 661M5 |
| Heart failure self-management plan review | 661N5 |
| New York Heart Association Classification - Class I | 662f |
| New York Heart Association Classification - Class II | 662g |
| New York Heart Association Classification - Class III | 662h |
| New York Heart Association Classification - Class IV | 662i |
| Heart failure 6 month review | 662p |
| Congestive heart failure monitoring | 662T |
| Heart failure annual review | 662W |
| Heart failure education | 679X |
| Cardiac failure therapy | 8B29 |
| Heart failure care plan discussed with patient | 8CL3 |
| Admit heart failure emergency | 8H2S |
| Heart failure follow-up | 8HBE |
| Discharge from practice nurse heart failure clinic | 8Hg8 |
| Referral to heart failure exercise programme | 8HHz |
| Referred to heart failure education group | 8Hk0 |
| Exception reporting: LVD quality indicators | 9h1 |
| Excepted from LVD quality indicators: Patient unsuitable | 9h11 |
| Excepted from LVD quality indicators: Informed dissent | 9h12 |
| Exception reporting: heart failure quality indicators | 9hH |
| Excepted heart failure quality indicators: Patient unsuitabl | 9hH0 |
| Excepted heart failure quality indicators: Informed dissent | 9hH1 |
| Seen in heart failure clinic | 9N0k |
| Seen by community heart failure nurse | 9N2p |
| Did not attend practice nurse heart failure clinic | 9N4s |
| Did not attend heart failure clinic | 9N4w |
| Referred by heart failure nurse specialist | 9N6T |
| Left ventricular dysfunction monitoring administration | 9On |
| Left ventricular dysfunction monitoring first letter | 9On0 |
| Left ventricular dysfunction monitoring second letter | 9On1 |
| Left ventricular dysfunction monitoring third letter | 9On2 |
| Left ventricular dysfunction monitoring verbal invite | 9On3 |
| Left ventricular dysfunction monitoring telephone invite | 9On4 |
| Heart failure monitoring administration | 9Or |
| Heart failure review completed | 9Or0 |
| Heart failure monitoring telephone invite | 9Or1 |
| Heart failure monitoring verbal invite | 9Or2 |
| Heart failure monitoring first letter | 9Or3 |
| Heart failure monitoring second letter | 9Or4 |
| Heart failure monitoring third letter | 9Or5 |
| Rheumatic left ventricular failure | G1yz1 |
| Malignant hypertensive heart disease | G210 |
| Malignant hypertensive heart disease with CCF | G2101 |
| Malignant hypertensive heart disease NOS | G210z |
| Benign hypertensive heart disease with CCF | G2111 |
| Hypertensive heart disease NOS with CCF | G21z1 |
| Hypertensive heart AND renal disease | G23 |
| Malignant hypertensive heart AND renal disease | G230 |
| Hypertensive heart&renal dis wth (congestive) heart failure | G232 |
| Hyperten heart&renal dis+both(congestv)heart and renal fail | G234 |
| Acute cor pulmonale | G400 |
| Acute pulmonary heart disease NOS | G40z |
| Chronic cor pulmonale | G41z-1 |
| Congestive cardiomyopathy | G5540 |
| Congestive obstructive cardiomyopathy | G5540-1 |
| Heart failure | G58 |
| Cardiac failure | G58-1 |
| Congestive heart failure | G580 |
| Congestive cardiac failure | G580-1 |
| Right heart failure | G580-2 |
| Right ventricular failure | G580-3 |
| Biventricular failure | G580-4 |
| Acute congestive heart failure | G5800 |
| Chronic congestive heart failure | G5801 |
| Decompensated cardiac failure | G5802 |
| Compensated cardiac failure | G5803 |
| Congestive heart failure due to valvular disease | G5804 |
| Left ventricular failure | G581 |
| Asthma - cardiac | G581-1 |
| Pulmonary oedema - acute | G581-2 |
| Impaired left ventricular function | G581-3 |
| Acute left ventricular failure | G5810 |
| Acute heart failure | G582 |
| Heart failure with normal ejection fraction | G583 |
| HFNEF - heart failure with normal ejection fraction | G583-1 |
| Heart failure with preserved ejection fraction | G583-2 |
| Right ventricular failure | G584 |
| Heart failure NOS | G58z |
| Cardiac failure NOS | G58z-2 |
| Left ventricular systolic dysfunction | G5yy9 |
| Left ventricular diastolic dysfunction | G5yyA |
| Pulmonary congestion and hypostasis | H54 |
| Pulmonary congestion | H541 |
| Chronic pulmonary oedema | H5410 |
| Pulmonary oedema NOS | H541z |
| Pulmonary congestion and hypostasis NOS | H54z |
| Acute pulmonary oedema unspecified | H584 |
| Acute pulmonary oedema NOS | H584z |
| Congenital cardiac failure | Q48y1 |
| [D]Cardiorespiratory failure | R2y10 |
| Heart failure as a complication of care | SP111-1 |
| AURAS-AF - consider the patient to have heart failure | EMISNQAU116 |
| Emergency heart failure admission since last appointment | EMISNQEM10 |
| Heart failure clinical pathway protocol followed | EMISNQHE59 |
| Heart failure information starter pack provided | EMISNQHE14 |
| Heart failure lifestyle plan commenced | EMISNQHE13 |
| Heart failure monitoring - co-medications | EMISNQHE20 |
| Heart failure monitoring - co-morbidities | EMISNQHE21 |
| Heart failure monitoring - multiple readmissions | EMISNQHE19 |
| Heart failure monitoring - palliative care | EMISNQHE22 |
| Heart failure monitoring - psychological issues | EMISNQHE18 |
| Heart failure monitoring - social issues | EMISNQHE17 |
| Heart failure monitoring - specialist clinical needs | EMISNQHE16 |
| Heart failure monitoring - unstable symptoms | EMISNQHE15 |
| Heart failure monitoring default | EMISNQHE72 |
| Heart failure monitoring in primary care | EMISNQHE70 |
| Heart failure monitoring in secondary care | EMISNQHE71 |
| Heart failure pathway protocol not followed | EMISNQHE58 |
| Heart failure resolved | EMISNQHE42 |
| Severe left ventricular systolic dysfunction | EMISNQSE142 |
| [RFC] Cardiac failure | HNG0013 |
| H/O: myocardial infarct <60 | 14A3 |
| H/O: myocardial infarct >60 | 14A4 |
| H/O: Myocardial infarction in last year | 14AH |
| History of myocardial infarction | 14AT |
| ECG: myocardial infarction | 323 |
| ECG: old myocardial infarction | 3232 |
| ECG: antero-septal infarct. | 3233 |
| ECG:posterior/inferior infarct | 3234 |
| ECG: subendocardial infarct | 3235 |
| ECG: lateral infarction | 3236 |
| ECG: myocardial infarct NOS | 323Z |
| Diab mellit insulin-glucose infus acute myocardial infarct | 889A |
| Acute myocardial infarction | G30 |
| Attack - heart | G30-1 |
| Coronary thrombosis | G30-2 |
| Cardiac rupture following myocardial infarction (MI) | G30-3 |
| Heart attack | G30-4 |
| MI - acute myocardial infarction | G30-5 |
| Thrombosis - coronary | G30-6 |
| Silent myocardial infarction | G30-7 |
| Acute anterolateral infarction | G300 |
| Other specified anterior myocardial infarction | G301 |
| Acute anteroapical infarction | G3010 |
| Acute anteroseptal infarction | G3011 |
| Anterior myocardial infarction NOS | G301z |
| Acute inferolateral infarction | G302 |
| Acute inferoposterior infarction | G303 |
| Posterior myocardial infarction NOS | G304 |
| Lateral myocardial infarction NOS | G305 |
| True posterior myocardial infarction | G306 |
| Acute subendocardial infarction | G307 |
| Acute non-Q wave infarction | G3070 |
| Acute non-ST segment elevation myocardial infarction | G3071 |
| Inferior myocardial infarction NOS | G308 |
| Acute Q-wave infarct | G309 |
| Acute posterolateral myocardial infarction | G30B |
| Acute transmural myocardial infarction of unspecif site | G30X |
| Acute ST segment elevation myocardial infarction | G30X0 |
| Other acute myocardial infarction | G30y |
| Acute atrial infarction | G30y0 |
| Acute papillary muscle infarction | G30y1 |
| Acute septal infarction | G30y2 |
| Other acute myocardial infarction NOS | G30yz |
| Acute myocardial infarction NOS | G30z |
| Postmyocardial infarction syndrome | G310 |
| Dressler's syndrome | G310-1 |
| Acute coronary syndrome | G3115 |
| Microinfarction of heart | G31y1 |
| Old myocardial infarction | G32 |
| Healed myocardial infarction | G32-1 |
| Personal history of myocardial infarction | G32-2 |
| Post infarct angina | G33z5 |
| Subsequent myocardial infarction | G35 |
| Subsequent myocardial infarction of anterior wall | G350 |
| Subsequent myocardial infarction of inferior wall | G351 |
| Subsequent myocardial infarction of other sites | G353 |
| Subsequent myocardial infarction of unspecified site | G35X |
| Certain current complication follow acute myocardial infarct | G36 |
| Haemopericardium/current comp folow acut myocard infarct | G360 |
| Atrial septal defect/curr comp folow acut myocardal infarct | G361 |
| Ventric septal defect/curr comp fol acut myocardal infarctn | G362 |
| Ruptur cardiac wall w'out haemopericard/cur comp fol ac MI | G363 |
| Ruptur chordae tendinae/curr comp fol acute myocard infarct | G364 |
| Rupture papillary muscle/curr comp fol acute myocard infarct | G365 |
| Thrombosis atrium,auric append&vent/curr comp foll acute MI | G366 |
| Postoperative myocardial infarction | G38 |
| Postoperative transmural myocardial infarction anterior wall | G380 |
| Postoperative transmural myocardial infarction inferior wall | G381 |
| Postoperative transmural myocardial infarction unspec site | G383 |
| Postoperative subendocardial myocardial infarction | G384 |
| Postoperative myocardial infarction, unspecified | G38z |
| Post infarction pericarditis | G501 |
| [X]Acute transmural myocardial infarction of unspecif site | Gyu34 |
| [X]Subsequent myocardial infarction of other sites | Gyu35 |
| [X]Subsequent myocardial infarction of unspecified site | Gyu36 |
| [RFC] Myocardial infarction (MI) | HNG0009 |
| Cause of Death- Acute Myocardial Infarction | EGTON2G41 |
| Cause of Death- Myocardial Infarction |  |
| First myocardial infarction | EMISR4QFI1 |
| H/O: aortic aneurysm | 14AE |
| H/O: Peripheral vascular disease procedure | 14NB |
| Ischaemic toe | 2G63 |
| Femoral arteriogram abnormal | 5593 |
| Lower limb arteriogram abnorm. | 55A2 |
| Aortic aneurysm monitoring | 66f3 |
| Emerg aortic bypass by anastomosis axillary to femoral art | 7A100 |
| Bypass aorta by anastomosis axillary to femoral artery NEC | 7A101 |
| Axillo-bifemoral bypass graft | 7A102 |
| Axillo-unifemoral PTFE bypass graft | 7A103 |
| Emerg repl aneurysm bifurc aorta by anast aorta to fem art | 7A110 |
| Emerg repl aneurysm bifurc aorta by anast aorta to iliac a | 7A112 |
| Y graft of abdominal Aortic aneurysm (emergency) | 7A112-1 |
| Other bypass of bifurcation of aorta | 7A12 |
| Emerg bypass bifurc aorta by anast aorta to femoral artery | 7A120 |
| Bypass bifurc aorta by anastom aorta to femoral artery NEC | 7A121 |
| Aorto bifemoral graft | 7A121-1 |
| Dacron aortofemoral Y graft | 7A121-2 |
| Bypass bifurcation aorta by anastom aorta to iliac artery | 7A123 |
| Aorto biiliac graft | 7A123-1 |
| Dacron aortoiliac Y graft | 7A123-2 |
| Other specified other bypass of bifurcation of aorta | 7A12y |
| Other bypass of bifurcation of aorta NOS | 7A12z |
| Emergency replacement of aneurysmal segment of aorta | 7A13 |
| Emergency repair of aortic aneurysm | 7A13-1 |
| Emerg replace aneurysm asc aorta by anastom aorta to aorta | 7A130 |
| Emerg replace aneurysm thor aorta by anastom aorta to aorta | 7A131 |
| Emerg replace aneurysm infrarenal aorta by anast aorta/aorta | 7A133 |
| Emerg replace aneurysm abdom aorta by anast aorta/aorta NEC | 7A134 |
| Tube graft abdominal Aortic aneurysm (emergency) | 7A134-1 |
| Emergency replacement of aneurysmal segment of aorta OS | 7A13y |
| Emergency replacement of aneurysmal segment of aorta NOS | 7A13z |
| Open embolectomy of bifurcation of aorta | 7A192 |
| Other bypass of iliac artery | 7A41 |
| Other bypass of iliac artery by anastomosis | 7A41-1 |
| Emerg bypass iliac art by iliac/femoral art anastomosis NEC | 7A410 |
| Bypass iliac artery by iliac/femoral artery anastomosis NEC | 7A411 |
| Emerg bypass iliac artery by femoral/femoral art anast NEC | 7A412 |
| Emergency femoro-femoral prosthetic cross over graft | 7A412-1 |
| Bypass iliac artery by femoral/femoral art anastomosis NEC | 7A413 |
| Femoro-femoral prosthetic cross over graft | 7A413-1 |
| Emerg bypass comm iliac art by aorta/com iliac art anast NEC | 7A414 |
| Emerg bypass leg artery by aorta/com fem art anastomosis NEC | 7A416 |
| Bypass common iliac artery by aorta/com iliac art anast NEC | 7A419 |
| Bypass leg artery by aorta/com femoral art anastomosis NEC | 7A41B |
| Bypass leg artery by aorta/deep femoral art anastomosis NEC | 7A41C |
| Bypass iliac artery by iliac/iliac artery anastomosis NEC | 7A41D |
| Emergency bypass of iliac artery by unspecified anastomosis | 7A41E |
| Ilio-femoral prosthetic cross over graft | 7A41F |
| Other specified other bypass of iliac artery | 7A41y |
| Other bypass of iliac artery NOS | 7A41z |
| Reconstruction of iliac artery | 7A42 |
| Reconstruction of common iliac artery | 7A42-1 |
| Endarterectomy and patch repair of iliac artery | 7A420 |
| Endarterectomy and patch repair of common iliac artery | 7A420-1 |
| Iliac endarterectomy and patch | 7A420-2 |
| Endarterectomy of iliac artery NEC | 7A421 |
| Endarterectomy of common iliac artery NEC | 7A421-1 |
| Other specified reconstruction of iliac artery | 7A42y |
| Reconstruction of iliac artery NOS | 7A42z |
| Other open operations on iliac artery | 7A43 |
| Other open operations on common iliac artery | 7A43-1 |
| Repair of iliac artery NEC | 7A430 |
| Repair of common iliac artery NEC | 7A430-1 |
| Open embolectomy of iliac artery | 7A431 |
| Open embolectomy of common iliac artery | 7A431-1 |
| Open insertion of iliac artery stent | 7A433 |
| Percutaneous transluminal angioplasty of iliac artery | 7A440 |
| Percutaneous transluminal embolectomy of iliac artery | 7A441 |
| Insertion of iliac artery stent | 7A443 |
| Percutaneous transluminal insertion of iliac artery stent | 7A444 |
| Other specified transluminal operation on iliac artery | 7A44y |
| Transluminal operation on iliac artery NOS | 7A44z |
| Other emergency bypass of femoral artery or popliteal artery | 7A47 |
| Other emerg bypass femoral or popliteal art by anastomosis | 7A47-1 |
| Other emergency bypass of common femoral artery | 7A47-2 |
| Other emergency bypass of deep femoral artery | 7A47-3 |
| Other emergency bypass of popliteal artery | 7A47-4 |
| Other emergency bypass of superficial femoral artery | 7A47-5 |
| Other emergency bypass of femoral artery | 7A47-6 |
| Emerg bypass femoral art by fem/pop art anast c prosth NEC | 7A470 |
| Emerg bypass popliteal art by pop/pop art anast c prosth NEC | 7A471 |
| Emerg bypass femoral art by fem/pop a anast c vein graft NEC | 7A472 |
| Emerg bypass pop art by pop/pop art anast c vein graft NEC | 7A473 |
| Emerg bypass femoral art by fem/tib art anast c prosth NEC | 7A474 |
| Emerg bypass femoral art by fem/tib a anast c vein graft NEC | 7A476 |
| Emerg bypass pop art by pop/tib art anast c vein graft NEC | 7A477 |
| Emerg bypass popliteal art by pop/peron a anast c prosth NEC | 7A479 |
| Emerg bypass fem art by fem/peron a anast c vein graft NEC | 7A47A |
| Emerg bypass pop art by pop/peron art anast c vein graft NEC | 7A47B |
| Emerg bypass femoral artery by fem/fem art anastomosis NEC | 7A47C |
| Emerg bypass popliteal artery by pop/fem art anastomosis NEC | 7A47D |
| Other emergency bypass of femoral or popliteal artery OS | 7A47y |
| Other emergency bypass of femoral or popliteal artery NOS | 7A47z |
| Other bypass of femoral artery or popliteal artery | 7A48 |
| Other bypass of femoral or popliteal artery by anastomosis | 7A48-1 |
| Other bypass of common femoral artery | 7A48-2 |
| Other bypass of femoral artery | 7A48-4 |
| Other bypass of popliteal artery | 7A48-5 |
| Other bypass of superficial femoral artery | 7A48-6 |
| Bypass femoral artery by fem/pop art anast c prosthesis NEC | 7A480 |
| Bypass popliteal artery by pop/pop a anast c prosthesis NEC | 7A481 |
| Bypass femoral artery by fem/pop art anast c vein graft NEC | 7A482 |
| Bypass popliteal artery by pop/pop a anast c vein graft NEC | 7A483 |
| Bypass femoral artery by fem/tib art anast c prosthesis NEC | 7A484 |
| Bypass popliteal artery by pop/tib a anast c prosthesis NEC | 7A485 |
| Bypass femoral artery by fem/tib art anast c vein graft NEC | 7A486 |
| Bypass popliteal artery by pop/tib a anast c vein graft NEC | 7A487 |
| Bypass femoral artery by fem/peron a anast c prosthesis NEC | 7A488 |
| Bypass popliteal artery by pop/peron art anast c prosth NEC | 7A489 |
| Bypass femoral artery by fem/peron a anast c vein graft NEC | 7A48A |
| Bypass popliteal art by pop/peron art anast c vein graft NEC | 7A48B |
| Bypass femoral artery by femoral/femoral art anastomosis NEC | 7A48C |
| Bypass popliteal artery by pop/fem artery anastomosis NEC | 7A48D |
| Femoro-femoral prosthetic cross over graft | 7A48E |
| Other bypass of femoral artery or popliteal artery OS | 7A48y |
| Other bypass of femoral artery or popliteal artery NOS | 7A48z |
| Reconstruction of femoral artery or popliteal artery | 7A49 |
| Reconstruction of common femoral artery | 7A49-1 |
| Reconstruction of deep femoral artery | 7A49-2 |
| Reconstruction of femoral artery | 7A49-3 |
| Reconstruction of popliteal artery | 7A49-4 |
| Reconstruction of superficial femoral artery | 7A49-5 |
| Endarterectomy and patch repair of femoral artery | 7A490 |
| Endarterectomy and patch repair of popliteal artery | 7A491 |
| Endarterectomy of femoral artery NEC | 7A492 |
| Endarterectomy of popliteal artery NEC | 7A493 |
| Profundoplasty femoral artery & patch repair deep fem artery | 7A494 |
| Profundoplasty and patch repair of popliteal artery | 7A495 |
| Profundoplasty of femoral artery NEC | 7A496 |
| Profundoplasty of popliteal artery NEC | 7A497 |
| Reconstruction of femoral artery with vein graft | 7A498 |
| Reconstruction of popliteal artery with vein graft | 7A499 |
| Reconstruction of femoral or popliteal artery OS | 7A49y |
| Reconstruction of femoral or popliteal artery NOS | 7A49z |
| Other open operations on femoral artery or popliteal artery | 7A4A |
| Other open operations on common femoral artery | 7A4A-1 |
| Other open operations on deep femoral artery | 7A4A-2 |
| Other open operations on popliteal artery | 7A4A-3 |
| Other open operations on superficial femoral artery | 7A4A-4 |
| Repair of femoral artery NEC | 7A4A0 |
| Repair of popliteal artery NEC | 7A4A1 |
| Open embolectomy of femoral artery | 7A4A2 |
| Open thrombectomy of femoral artery | 7A4A2-1 |
| Open femoral embolectomy | 7A4A2-2 |
| Open embolectomy popliteal artery | 7A4A3 |
| Open thrombectomy of popliteal artery | 7A4A3-1 |
| Ligation of aneurysm of popliteal artery | 7A4A4 |
| Operation on aneurysm of femoral artery NEC | 7A4A5 |
| Operation on popliteal artery NEC | 7A4A6 |
| Repair of femoral artery with temporary silastic shunt | 7A4A7 |
| Repair of popliteal artery with temporary silastic shunt | 7A4A8 |
| Other open operation on femoral or popliteal artery OS | 7A4Ay |
| Other open operation on femoral or popliteal artery NOS | 7A4Az |
| Percutaneous transluminal angioplasty of femoral artery | 7A4B0 |
| Percutaneous transluminal angioplasty of popliteal artery | 7A4B1 |
| Percutaneous transluminal embolectomy of femoral artery | 7A4B2 |
| Percutaneous transluminal embolectomy of popliteal artery | 7A4B3 |
| Percutaneous transluminal embolisation of femoral artery | 7A4B4 |
| Percutaneous transluminal embolisation of popliteal artery | 7A4B5 |
| Percut translum thrombolysis femoral graft streptokinase | 7A4B8 |
| Percutaneous transluminal insertion of stent femoral artery | 7A4B9 |
| Revision of reconstruction of artery | 7A50 |
| Revision of reconstruction involving aorta | 7A500 |
| Revision of reconstruction involving iliac artery | 7A501 |
| Revision of reconstruction involving femoral artery | 7A502 |
| Revision of reconstruction of popliteal artery | 7A503 |
| Other specified revision of reconstruction of artery | 7A50y |
| Revision of reconstruction of artery NOS | 7A50z |
| Gas gangrene-foot | A3A0F |
| Diabetes mellitus with peripheral circulatory disorder | C107 |
| Diabetes mellitus, juvenile +peripheral circulatory disorder | C1070 |
| Diabetes mellitus, adult, + peripheral circulatory disorder | C1071 |
| IDDM with peripheral circulatory disorder | C1073 |
| NIDDM with peripheral circulatory disorder | C1074 |
| Other specified diabetes mellitus with periph circ comps | C107y |
| Diabetes mellitus NOS with peripheral circulatory disorder | C107z |
| Insulin dependent diab mell with peripheral angiopathy | C108G |
| Non-insulin-dependent d m with peripheral angiopath | C109F |
| Type II diabetes mellitus with peripheral angiopathy | C109F-1 |
| Type 2 diabetes mellitus with peripheral angiopathy | C109F-2 |
| Type 1 diabetes mellitus with peripheral angiopathy | C10EG |
| Type 2 diabetes mellitus with peripheral angiopathy | C10FF |
| Aorto-iliac disease | G700-1 |
| Extremity artery atheroma | G702 |
| Extremity artery atheroma NOS | G702z |
| Aortic aneurysm | G71 |
| Abdominal aortic aneurysm which has ruptured | G713 |
| Ruptured abdominal aortic aneurysm | G713-1 |
| Ruptured suprarenal aortic aneurysm | G7130 |
| Abdominal aortic aneurysm without mention of rupture | G714 |
| AAA - Abdominal aortic aneurysm without mention of rupture | G714-1 |
| Juxtarenal aortic aneurysm | G7140 |
| Ruptured aortic aneurysm NOS | G715 |
| Thoracoabdominal aortic aneurysm, ruptured | G7150 |
| Aortic aneurysm without mention of rupture NOS | G716 |
| Thoracoabdominal aortic aneurysm, without mention of rupture | G7160 |
| Leaking abdominal aortic aneurysm | G718 |
| Aortic aneurysm NOS | G71z |
| Other peripheral vascular disease | G73 |
| Peripheral ischaemic vascular disease | G73-1 |
| Ischaemia of legs | G73-2 |
| Peripheral ischaemia | G73-3 |
| Thromboangiitis obliterans | G731 |
| Buerger's disease | G7310 |
| Thromboangiitis obliterans NOS | G731z |
| Peripheral gangrene | G732 |
| Gangrene of toe | G7320 |
| Gangrene of foot | G7321 |
| Ischaemic foot | G733 |
| Other specified peripheral vascular disease | G73y |
| Diabetic peripheral angiopathy | G73y0 |
| Peripheral angiopathic disease EC NOS | G73y1 |
| Other specified peripheral vascular disease NOS | G73yz |
| Peripheral vascular disease NOS | G73z |
| Intermittent claudication | G73z0 |
| Claudication | G73z0-1 |
| Peripheral vascular disease NOS | G73zz |
| Aortoiliac obstruction | G740-2 |
| Embolism and thrombosis of the femoral artery | G7424 |
| Embolism and thrombosis of the popliteal artery | G7425 |
| Embolism and thrombosis of the anterior tibial artery | G7426 |
| Embolism and thrombosis of the dorsalis pedis artery | G7427 |
| Embolism and thrombosis of a leg artery NOS | G7429 |
| Peripheral arterial embolism and thrombosis NOS | G742z |
| Embolism and/or thrombosis of the common iliac artery | G74y0 |
| Embolism and/or thrombosis of the internal iliac artery | G74y1 |
| Embolism and/or thrombosis of the external iliac artery | G74y2 |
| Embolism and thrombosis of the iliac artery unspecified | G74y3 |
| [X]Other specified peripheral vascular diseases | Gyu74 |
| Ischaemic leg ulcer | M271-2 |
| Ischaemic ulcer diabetic foot | M2710 |
| Arterial leg ulcer | M2713 |
| Mixed venous and arterial leg ulcer | M2714 |
| [D]Gangrene of toe in diabetic | R0542 |
| [D]Widespread diabetic foot gangrene | R0543 |
| [D]Failure of peripheral circulation | R0550 |
| [D]Peripheral circulatory failure | R0550-1 |
| [RFC] Peripheral vascular disease | HNG0172 |
| Ischaemic foot | EMISNQIS4 |
| Peripheral vascular disease annual review | EMISNQPE9 |
| Peripheral vascular disease monitoring administration | EMISNQPE10 |
| Peripheral vascular disease monitoring first letter | EMISNQPE11 |
| Peripheral vascular disease monitoring second letter | EMISNQPE12 |
| Peripheral vascular disease monitoring third letter | EMISNQPE13 |
| Transluminal balloon angioplasty of coronary artery | 7928 |
| Percutaneous balloon coronary angioplasty | 7928-1 |
| Percut transluminal balloon angioplasty one coronary artery | 79280 |
| Percut translum balloon angioplasty mult coronary arteries | 79281 |
| Percut translum balloon angioplasty bypass graft coronary a | 79282 |
| Percut translum cutting balloon angioplasty coronary artery | 79283 |
| Transluminal balloon angioplasty of coronary artery OS | 7928y |
| Transluminal balloon angioplasty of coronary artery NOS | 7928z |
| Percutaneous transluminal laser coronary angioplasty | 79290 |
| Rotary blade coronary angioplasty | 79293 |
| Insertion of coronary artery stent | 79294 |
| Insertion of drug-eluting coronary artery stent | 79295 |
| Percutaneous transluminal atherectomy of coronary artery | 79296 |
| Endarterectomy of coronary artery NEC | 792B0 |
| Perc translumin balloon angioplasty stenting coronary artery | 793G |
| Perc translum ball angio insert 1-2 drug elut stents cor art | 793G0 |
| Perc tran ball angio ins 3 or more drug elut stents cor art | 793G1 |
| Perc translum balloon angioplasty insert 1-2 stents cor art | 793G2 |
| Percutaneous cor balloon angiop 3 more stents cor art NEC | 793G3 |
| OS perc translumina balloon angioplast stenting coronary art | 793Gy |
| Perc translum balloon angioplasty stenting coronary art NOS | 793Gz |
| Percutaneous transluminal angioplasty of artery NEC | 7A540 |
| Rotary blade angioplasty | 7A545 |
| Percutaneous transluminal atherectomy | 7A548 |
| Percutaneous transluminal balloon angioplasty of artery | 7A564 |
| Peroperative angioplasty | 7A6G1 |
| Prosthetic graft patch angioplasty | 7A6H3 |
| Percutaneous transluminal angioplasty of vascular graft | 7A6H4 |
| [V]Presence of coronary angioplasty implant and graft | ZV458 |
| [V]Status following coronary angioplasty NOS | ZV45L |
| Stroke group member | 13YA |
| H/O: CVA/stroke | 14A7 |
| H/O: CVA | 14A7-1 |
| H/O: stroke | 14A7-2 |
| H/O: TIA | 14AB |
| H/O: Stroke in last year | 14AK |
| Stroke self-management plan agreed | 661M7 |
| Stroke self-management plan review | 661N7 |
| Stroke/CVA annual review | 662e |
| Stroke annual review | 6.62E+01 |
| Stroke 6 month review | 662M1 |
| Stroke initial post discharge review | 662M2 |
| Haemorrhagic stroke monitoring | 662o |
| Evacuation of subdural haematoma | 70170 |
| Evacuation of extradural haematoma | 70320 |
| Delivery of rehabilitation for stroke | 7P242 |
| Stroke / transient ischaemic attack referral | 8HBJ |
| Ref to multidisciplinary stroke function improvement service | 8HHM |
| Exception reporting: stroke quality indicators | 9h2 |
| Excepted from stroke quality indicators: Patient unsuitable | 9h21 |
| Excepted from stroke quality indicators: Informed dissent | 9h22 |
| Rupture of syphilitic cerebral aneurysm | A94y6 |
| Mitochond encephalopathy, lact acidosis & strokelike episode | C3151 |
| [X]Other transient cerebral ischaemic attacks and related syndromes | Fyu55 |
| [X]Other lacunar syndromes | Fyu56 |
| [X]Other vascular syndroms/brain in cerebrovasculr diseases | Fyu57 |
| Subarachnoid haemorrhage | G60 |
| Ruptured berry aneurysm | G600 |
| Subarachnoid haemorrhage from carotid siphon and bifurcation | G601 |
| Subarachnoid haemorrhage from middle cerebral artery | G602 |
| Subarachnoid haemorrhage from anterior communicating artery | G603 |
| Subarachnoid haemorrhage from posterior communicating artery | G604 |
| Subarachnoid haemorrhage from basilar artery | G605 |
| Subarachnoid haemorrhage from vertebral artery | G606 |
| Subarachnoid haemorrh from intracranial artery, unspecif | G60X |
| Subarachnoid haemorrhage NOS | G60z |
| Intracerebral haemorrhage | G61 |
| CVA - cerebrovascular accid due to intracerebral haemorrhage | G61-1 |
| Stroke due to intracerebral haemorrhage | G61-2 |
| Cortical haemorrhage | G610 |
| Internal capsule haemorrhage | G611 |
| Basal nucleus haemorrhage | G612 |
| Cerebellar haemorrhage | G613 |
| Pontine haemorrhage | G614 |
| Bulbar haemorrhage | G615 |
| External capsule haemorrhage | G616 |
| Intracerebral haemorrhage, intraventricular | G617 |
| Intracerebral haemorrhage, multiple localized | G618 |
| Lobar cerebral haemorrhage | G619 |
| Intracerebral haemorrhage in hemisphere, unspecified | G61X |
| Left sided intracerebral haemorrhage, unspecified | G61X0 |
| Right sided intracerebral haemorrhage, unspecified | G61X1 |
| Intracerebral haemorrhage NOS | G61z |
| Other and unspecified intracranial haemorrhage | G62 |
| Extradural haemorrhage - nontraumatic | G620 |
| Subdural haemorrhage - nontraumatic | G621 |
| Subdural haematoma - nontraumatic | G622 |
| Subdural haemorrhage NOS | G623 |
| Intracranial haemorrhage NOS | G62z |
| Cerebral infarct due to thrombosis of precerebral arteries | G63y0 |
| Cerebral infarction due to embolism of precerebral arteries | G63y1 |
| Cerebral arterial occlusion | G64 |
| CVA - cerebral artery occlusion | G64-1 |
| Infarction - cerebral | G64-2 |
| Stroke due to cerebral arterial occlusion | G64-3 |
| Cerebral thrombosis | G640 |
| Cerebral infarction due to thrombosis of cerebral arteries | G6400 |
| Cerebral embolism | G641 |
| Cerebral embolus | G641-1 |
| Cerebral infarction due to embolism of cerebral arteries | G6410 |
| Cerebral infarction NOS | G64z |
| Brainstem infarction NOS | G64z-1 |
| Cerebellar infarction | G64z-2 |
| Brainstem infarction | G64z0 |
| Wallenberg syndrome | G64z1 |
| Lateral medullary syndrome | G64z1-1 |
| Left sided cerebral infarction | G64z2 |
| Right sided cerebral infarction | G64z3 |
| Infarction of basal ganglia | G64z4 |
| Transient cerebral ischaemia | G65 |
| Transient ischaemic attack | G65-2 |
| Carotid artery syndrome hemispheric | G653 |
| Multiple and bilateral precerebral artery syndromes | G654 |
| Carotid territory transient ischaemic attack | G657 |
| Other transient cerebral ischaemia | G65y |
| Transient cerebral ischaemia NOS | G65z |
| Impending cerebral ischaemia | G65z0 |
| Intermittent cerebral ischaemia | G65z1 |
| Transient cerebral ischaemia NOS | G65zz |
| Stroke and cerebrovascular accident unspecified | G66 |
| CVA unspecified | G66-1 |
| Stroke unspecified | G66-2 |
| CVA - Cerebrovascular accident unspecified | G66-3 |
| Middle cerebral artery syndrome | G660 |
| Anterior cerebral artery syndrome | G661 |
| Posterior cerebral artery syndrome | G662 |
| Brain stem stroke syndrome | G663 |
| Cerebellar stroke syndrome | G664 |
| Pure motor lacunar syndrome | G665 |
| Pure sensory lacunar syndrome | G666 |
| Left sided CVA | G667 |
| Right sided CVA | G668 |
| Generalised ischaemic cerebrovascular disease NOS | G671 |
| Acute cerebrovascular insufficiency NOS | G6710 |
| Chronic cerebral ischaemia | G6711 |
| Generalised ischaemic cerebrovascular disease NOS | G671z |
| Cereb infarct due cerebral venous thrombosis, nonpyogenic | G6760 |
| Late effects of cerebrovascular disease | G68 |
| Sequelae of subarachnoid haemorrhage | G680 |
| Sequelae of intracerebral haemorrhage | G681 |
| Sequelae of other nontraumatic intracranial haemorrhage | G682 |
| Sequelae of cerebral infarction | G683 |
| Sequelae/other + unspecified cerebrovascular diseases | G68W |
| Sequelae of stroke,not specfd as h'morrhage or infarction | G68X |
| Cereb infarct due unsp occlus/stenos precerebr arteries | G6W |
| Cerebrl infarctn due/unspcf occlusn or sten/cerebrl artrs | G6X |
| [X]Subarachnoid haemorrhage from other intracranial arteries | Gyu60 |
| [X]Other subarachnoid haemorrhage | Gyu61 |
| [X]Other intracerebral haemorrhage | Gyu62 |
| [X]Cerebrl infarctn due/unspcf occlusn or sten/cerebrl artrs | Gyu63 |
| [X]Other cerebral infarction | Gyu64 |
| [X]Occlusion and stenosis of other precerebral arteries | Gyu65 |
| [X]Occlusion and stenosis of other cerebral arteries | Gyu66 |
| [X]Sequelae of stroke,not specfd as h'morrhage or infarction | Gyu6C |
| [X]Subarachnoid haemorrh from intracranial artery, unspecif | Gyu6E |
| [X]Intracerebral haemorrhage in hemisphere, unspecified | Gyu6F |
| [X]Cereb infarct due unsp occlus/stenos precerebr arteries | Gyu6G |
| CVA - cerebrovascular accident in the puerperium | L440-1 |
| Stroke in the puerperium | L440-2 |
| [V]Personal history of stroke | ZV125-1 |
| [V]Personal history of cerebrovascular accident (CVA) | ZV125-2 |
| [RFC] Ischaemic attack | HNG0237 |
| [RFC] Stroke | HNG0235 |
| [RFC] Stroke | HNG0602 |
| [RFC] Stroke/CVA | HNG0234 |
| Central post-stroke pain | EMISNQCE10 |
| Discharge from community stroke service | EMISNQDI251 |
| Referral to community stroke service | EMISNQRE623 |
| Referral to stroke rehabilitation service | EMISNQRE602 |
| Suspected transient ischaemic attack | EMISNQSU26 |
| Cerebral infarction with haemorrhagic transformation | EMISNQCE4 |
| Cause of Death- Cerebral Infarct |  |
| [RFC] CVA | HNGP003 |
| H/O ventricular fibrillation | 14AD |
| O/E - collapse -cardiac arrest | 2241 |
| ECG: ventricular fibrillation | 3283 |
| Cardiac massage - open | 79321-1 |
| Advanced cardiopulmonary resuscitation | 7L1H6 |
| Cardiac massage - external | 853 |
| Closed cardiac massage alone | 8531 |
| Closed cardiac massage+ventil. | 8532 |
| Cardiopulmonary resuscitation | 8532-1 |
| External cardiac massage NOS | 853Z |
| Ventricular fibrillation and flutter | G574 |
| Ventricular fibrillation | G5740 |
| Cardiac arrest-ventricular fibrillation | G5740-1 |
| Ventricular fibrillation and flutter NOS | G574z |
| Cardiac arrest | G575 |
| Cardio-respiratory arrest | G575-1 |
| Asystole | G575-2 |
| Cardiac arrest with successful resuscitation | G5750 |
| Sudden cardiac death, so described | G5751 |
| Electromechanical dissociation with successful resuscitation | G5752 |
| Electromechanical dissociation | G5753 |
| Cardiac arrest, unspecified | G575z |
| Cardiac arrest as a complication of care | SP110 |
|  |  |
| **Term** | **ICD 10 code** |
| Angina pectoris | I20 |
| Unstable angina | I200 |
| Angina pectoris with documented spasm | I201 |
| Other forms of angina pectoris | I208 |
| Angina pectoris unspecified | I209 |
| Presence of coronary angioplasty implant and graft | Z955 |
| Other acute ischaemic heart diseases | I24 |
| Coronary thrombosis not resulting in myocardial infarction | I240 |
| Other forms of acute ischaemic heart disease | I248 |
| Acute ischaemic heart disease, unspecified | I249 |
| Chronic ischaemic heart disease | I25 |
| Atherosclerotic cardiovascular disease, so described | I250 |
| Atherosclerotic heart disease | I251 |
| Coronary artery aneurysm | I254 |
| Ischaemic cardiomyopathy | I255 |
| Silent myocardial ischaemia | I256 |
| Other forms of chronic ischaemic heart disease | I258 |
| Chronic ischaemic heart disease, unspecified | I259 |
| Hypertensive heart disease with (congestive) heart failure | I110 |
| Hypertensive heart and renal disease with (congestive) heart failure | I130 |
| Hypertensive heart and renal disease with both (congestive) heart failure and renal failure | I132 |
| Heart failure | I50 |
| Congestive heart failure | I500 |
| Left ventricular failure | I501 |
| Heart failure, unspecified | I509 |
| Acute myocardial infarction | I21 |
| Acute transmural myocardial infarction of anterior wall | I210 |
| Acute transmural myocardial infarction of inferior wall | I211 |
| Acute transmural myocardial infarction of other sites | I212 |
| Acute transmural myocardial infarction of unspecified site | I213 |
| Acute subendocardial myocardial infarction | I214 |
| Acute myocardial infarction, unspecified | I219 |
| Subsequent myocardial infarction | I22 |
| Subsequent myocardial infarction of anterior wall | I220 |
| Subsequent myocardial infarction of inferior wall | I221 |
| Subsequent myocardial infarction of other sites | I228 |
| Subsequent myocardial infarction of unspecified site | I229 |
| Certain current complications following acute myocardial infarction | I23 |
| Haemopericardium as current complication following acute myocardial infarction | I230 |
| Atrial septal defect as current complication following acute myocardial infarction | I231 |
| Ventricular septal defect as current complication following acute myocardial infarction | I232 |
| Rupture of cardiac wall without haemopericardium as current complication following acute myocardial infarction | I233 |
| Rupture of chordae tendineae as current complication following acute myocardial infarction | I234 |
| Rupture of papillary muscle as current complication following acute myocardial infarction | I235 |
| Thrombosis of atrium, auricular appendage, and ventricle as current complications following acute myocardial infarction | I236 |
| Other current complications following acute myocardial infarction | I238 |
| Dressler's syndrome | I241 |
| Old myocardial infarction | I252 |
| Abdominal aortic aneurysm, ruptured | I713 |
| Abdominal aortic aneurysm, without mention of rupture | I714 |
| Thoracoabdominal aortic aneurysm, ruptured | I715 |
| Thoracoabdominal aortic aneurysm, without mention of rupture | I716 |
| Aortic aneurysm of unspecified site, ruptured | I718 |
| Aortic aneurysm of unspecified site, without mention of rupture | I719 |
| Thromboangiitis obliterans [Buerger] | I731 |
| Other specified peripheral vascular diseases | I738 |
| Peripheral vascular disease, unspecified | I739 |
| Embolism and thrombosis of arteries of lower extremities | I743 |
| Embolism and thrombosis of arteries of extremities, unspecified | I744 |
| Embolism and thrombosis of iliac artery | I745 |
| Other transient cerebral ischaemic attacks and related syndromes | G458 |
| Transient cerebral ischaemic attack, unspecified | G459 |
| Middle cerebral artery syndrome | G460 |
| Anterior cerebral artery syndrome | G461 |
| Posterior cerebral artery syndrome | G462 |
| Brain stem stroke syndrome | G463 |
| Cerebellar stroke syndrome | G464 |
| Pure motor lacunar syndrome | G465 |
| Pure sensory lacunar syndrome | G466 |
| Other lacunar syndromes | G467 |
| Subarachnoid haemorrhage | I60 |
| Subarachnoid haemorrhage from carotid siphon and bifurcation | I600 |
| Subarachnoid haemorrhage from middle cerebral artery | I601 |
| Subarachnoid haemorrhage from anterior communicating artery | I602 |
| Subarachnoid haemorrhage from posterior communicating artery | I603 |
| Subarachnoid haemorrhage from basilar artery | I604 |
| Subarachnoid haemorrhage from vertebral artery | I605 |
| Subarachnoid haemorrhage from other intracranial arteries | I606 |
| Subarachnoid haemorrhage from intracranial artery, unspecified | I607 |
| Other subarachnoid haemorrhage | I608 |
| Subarachnoid haemorrhage, unspecified | I609 |
| Intracerebral haemorrhage | I61 |
| Intracerebral haemorrhage in hemisphere, subcortical | I610 |
| Intracerebral haemorrhage in hemisphere, cortical | I611 |
| Intracerebral haemorrhage in hemisphere, unspecified | I612 |
| Intracerebral haemorrhage in brain stem | I613 |
| Intracerebral haemorrhage in cerebellum | I614 |
| Intracerebral haemorrhage, intraventricular | I615 |
| Intracerebral haemorrhage, multiple localized | I616 |
| Other intracerebral haemorrhage | I618 |
| Intracerebral haemorrhage, unspecified | I618 |
| Nontraumatic intracerebral hemorrhage, unspecified | I619 |
| Other nontraumatic intracranial haemorrhage | I62 |
| Subdural haemorrhage (acute)(nontraumatic) | I620 |
| Nontraumatic extradural haemorrhage | I621 |
| Intracranial haemorrhage (nontraumatic), unspecified | I629 |
| Cerebral infarction | I63 |
| Cerebral infarction due to thrombosis of precerebral arteries | I630 |
| Cerebral infarction due to embolism of precerebral arteries | I631 |
| Cerebral infarction due to unspecified occlusion or stenosis of precerebral arteries | I632 |
| Cerebral infarction due to thrombosis of cerebral arteries | I633 |
| Cerebral infarction due to embolism of cerebral arteries | I634 |
| Cerebral infarction due to unspecified occlusion or stenosis of cerebral arteries | I635 |
| Cerebral infarction due to cerebral venous thrombosis, nonpyogenic | I636 |
| Other cerebral infarction | I638 |
| Cerebral infarction, unspecified | I639 |
| Stroke, not specified as haemorrhage or infarction | I64 |
| Sequelae of subarachnoid haemorrhage | I690 |
| Sequelae of intracerebral haemorrhage | I691 |
| Sequelae of other nontraumatic intracranial haemorrhage | I692 |
| Sequelae of cerebral infarction | I693 |
| Sequelae of stroke, not specified as haemorrhage or infarction | I694 |
| Sequelae of other and unspecified cerebrovascular diseases | I698 |
| Cardiac arrest | I46 |
| Cardiac arrest with successful resuscitation | I460 |
| Sudden cardiac death, so described | I461 |
| Cardiac arrest, unspecified | I469 |
| Re-entry ventricular arrhythmia | I470 |
| Ventricular fibrillation and flutter | I490 |
|  |  |
| **TERM** | **OPCS code** |
| Saphenous vein graft replacement of coronary artery | K40 |
| Saphenous vein graft replacement of one coronary artery | K40.1 |
| Saphenous vein graft replacement of two coronary arteries | K40.2 |
| Saphenous vein graft replacement of three coronary arteries | K40.3 |
| Saphenous vein graft replacement of four or more coronary arteries | K40.4 |
| Other specified saphenous vein graft replacement of coronary artery | K40.8 |
| Unspecified saphenous vein graft replacement of coronary artery | K40.9 |
| Other autograft replacement of coronary artery | K41 |
| Autograft replacement of one coronary artery NEC | K41.1 |
| Autograft replacement of two coronary arteries NEC | K41.2 |
| Autograft replacement of three coronary arteries NEC | K41.3 |
| Autograft replacement of four or more coronary arteries NEC | K41.4 |
| Other specified other autograft replacement of coronary artery | K41.8 |
| Unspecified other autograft replacement of coronary artery | K41.9 |
| Allograft replacement of coronary artery | K42 |
| Allograft replacement of one coronary artery | K42.1 |
| Allograft replacement of two coronary arteries | K42.2 |
| Allograft replacement of three coronary arteries | K42.3 |
| Allograft replacement of four or more coronary arteries | K42.4 |
| Other specified allograft replacement of coronary artery | K42.8 |
| Unspecified allograft replacement of coronary artery | K42.9 |
| Prosthetic replacement of coronary artery | K43 |
| Prosthetic replacement of one coronary artery | K43.1 |
| Prosthetic replacement of two coronary arteries | K43.2 |
| Prosthetic replacement of three coronary arteries | K43.3 |
| Prosthetic replacement of four or more coronary arteries | K43.4 |
| Other specified prosthetic replacement of coronary artery | K43.8 |
| Unspecified prosthetic replacement of coronary artery | K43.9 |
| Other replacement of coronary artery | K44 |
| Replacement of coronary arteries using multiple methods | K44.1 |
| Revision of replacement of coronary artery | K44.2 |
| Other specified other replacement of coronary artery | K44.8 |
| Unspecified other replacement of coronary artery | K44.9 |
| Connection of thoracic artery to coronary artery | K45 |
| Double anastomosis of mammary arteries to coronary arteries | K45.1 |
| Double anastomosis of thoracic arteries to coronary arteries NEC | K45.2 |
| Anastomosis of mammary artery to left anterior descending coronary artery | K45.3 |
| Anastomosis of mammary artery to coronary artery NEC | K45.4 |
| Anastomosis of thoracic artery to coronary artery NEC | K45.5 |
| Revision of connection of thoracic artery to coronary artery | K45.6 |
| Other specified connection of thoracic artery to coronary artery | K45.8 |
| Unspecified connection of thoracic artery to coronary artery | K45.9 |
| Other bypass of coronary artery | K46 |
| Double implantation of mammary arteries into heart | K46.1 |
| Double implantation of thoracic arteries into heart NEC | K46.2 |
| Implantation of mammary artery into heart NEC | K46.3 |
| Implantation of thoracic artery into heart NEC | K46.4 |
| Revision of implantation of thoracic artery into heart | K46.5 |
| Other specified other bypass of coronary artery | K46.8 |
| Unspecified other bypass of coronary artery | K46.9 |
| Emergency replacement of aneurysmal segment of aorta | L18 |
| Emergency replacement of aneurysmal segment of ascending aorta by anastomosis of aorta to aorta | L181 |
| Emergency replacement of aneurysmal segment of thoracic aorta by anastomosis of aorta to aorta NEC | L182 |
| Emergency replacement of aneurysmal segment of suprarenal abdominal aorta by anastomosis of aorta to aorta | L183 |
| Emergency replacement of aneurysmal segment of infrarenal abdominal aorta by anastomosis of aorta to aorta | L184 |
| Emergency replacement of aneurysmal segment of abdominal aorta by anastomosis of aorta to aorta NEC | L185 |
| Emergency replacement of aneurysmal bifurcation of aorta by anastomosis of aorta to iliac artery | L186 |
| Other specified emergency replacement of aneurysmal segment of aorta | L188 |
| Unspecified emergency replacement of aneurysmal segment of aorta | L189 |
| Other replacement of aneurysmal segment of aorta | L19 |
| Replacement of aneurysmal segment of ascending aorta by anastomosis of aorta to aorta NEC | L191 |
| Replacement of aneurysmal segment of thoracic aorta by anastomosis of aorta to aorta NEC | L192 |
| Replacement of aneurysmal segment of suprarenal abdominal aorta by anastomosis of aorta to aorta NEC | L193 |
| Replacement of aneurysmal segment of infrarenal abdominal aorta by anastomosis of aorta to aorta NEC | L194 |
| Replacement of aneurysmal segment of abdominal aorta by anastomosis of aorta to aorta NEC | L195 |
| Replacement of aneurysmal bifurcation of aorta by anastomosis of aorta to iliac artery NEC | L196 |
| Other specified other replacement of aneurysmal segment of aorta | L198 |
| Unspecified other replacement of aneurysmal segment of aorta | L199 |
| Other emergency bypass of segment of aorta | L20 |
| Emergency bypass of segment of ascending aorta by anastomosis of aorta to aorta NEC | L201 |
| Emergency bypass of segment of thoracic aorta by anastomosis of aorta to aorta NEC | L202 |
| Emergency bypass of segment of suprarenal abdominal aorta by anastomosis of aorta to aorta NEC | L203 |
| Emergency bypass of segment of infrarenal abdominal aorta by anastomosis of aorta to aorta NEC | L204 |
| Emergency bypass of segment of abdominal aorta by anastomosis of aorta to aorta NEC | L205 |
| Emergency bypass of bifurcation of aorta by anastomosis of aorta to iliac artery NEC | L206 |
| Other specified other emergency bypass of segment of aorta | L208 |
| Unspecified other emergency bypass of segment of aorta | L209 |
| Operations on aneurysm of aorta NEC | L254 |
| Transluminal insertion of stent graft for aneurysmal segment of aorta | L27 |
| Endovascular insertion of stent graft for infrarenal abdominal aortic aneurysm | L271 |
| Endovascular insertion of stent graft for suprarenal aortic aneurysm | L272 |
| Endovascular insertion of stent graft for thoracic aortic aneurysm | L273 |
| Endovascular insertion of stent graft for aortic dissection in any position | L274 |
| Endovascular insertion of stent graft for aortic aneurysm of bifurcation NEC | L275 |
| Endovascular insertion of stent graft for aorto-uniiliac aneurysm | L276 |
| Other specified transluminal insertion of stent graft for aneurysmal segment of aorta | L278 |
| Unspecified transluminal insertion of stent graft for aneurysmal segment of aorta | L279 |
| Transluminal operations on aneurysmal segment of aorta | L28 |
| Endovascular insertion of stent for infrarenal abdominal aortic aneurysm | L281 |
| Endovascular insertion of stent for suprarenal aortic aneurysm | L282 |
| Endovascular insertion of stent for thoracic aortic aneurysm | L283 |
| Endovascular insertion of stent for aortic dissection in any position | L284 |
| Endovascular insertion of stent for aortic aneurysm of bifurcation NEC | L285 |
| Endovascular insertion of stent for aorto-uniiliac aneurysm | L286 |
| Other specified transluminal operations on aneurysmal segment of aorta | L288 |
| Unspecified transluminal operations on aneurysmal segment of aort | L289 |
| Other emergency bypass of iliac artery | L50 |
| Emergency bypass of common iliac artery by anastomosis of aorta to common iliac artery NEC | L50.1 |
| Emergency bypass of iliac artery by anastomosis of aorta to external iliac artery NEC | L50.2 |
| Emergency bypass of artery of leg by anastomosis of aorta to common femoral artery NEC | L50.3 |
| Emergency bypass of artery of leg by anastomosis of aorta to deep femoral artery NEC | L50.4 |
| Emergency bypass of iliac artery by anastomosis of iliac artery to iliac artery NEC | L50.5 |
| Emergency bypass of artery of leg by anastomosis of iliac artery to femoral artery NEC | L50.6 |
| Other specified other emergency bypass of iliac artery | L50.8 |
| Unspecified other emergency bypass of iliac artery | L50.9 |
| Other bypass of iliac artery | L51 |
| Bypass of common iliac artery by anastomosis of aorta to common iliac artery NEC | L51.1 |
| Bypass of iliac artery by anastomosis of aorta to external iliac artery NEC | L51.2 |
| Bypass of artery of leg by anastomosis of aorta to common femoral artery NEC | L51.3 |
| Bypass of artery of leg by anastomosis of aorta to deep femoral artery NEC | L51.4 |
| Bypass of iliac artery by anastomosis of iliac artery to iliac artery NEC | L51.5 |
| Bypass of artery of leg by anastomosis of iliac artery to femoral artery NEC | L51.6 |
| Other specified other bypass of iliac artery | L51.8 |
| Unspecified other bypass of iliac artery | L51.9 |
| Reconstruction of iliac artery | L52 |
| Endarterectomy of iliac artery and patch repair of iliac artery | L52.1 |
| Endarterectomy of iliac artery NEC | L52.2 |
| Other specified reconstruction of iliac artery | L52.8 |
| Unspecified reconstruction of iliac artery | L52.9 |
| Other open operations on iliac artery | L53 |
| Repair of iliac artery NEC | L53.1 |
| Open embolectomy of iliac artery | L53.2 |
| Percutaneous transluminal angioplasty of iliac artery | L54.1 |
| Percutaneous transluminal embolectomy of iliac artery | L54.2 |
| Percutaneous transluminal insertion of stent into iliac artery | L54.4 |
| Other specified transluminal operations on iliac artery | L54.8 |
| Unspecified transluminal operations on iliac artery | L54.9 |
| Other emergency bypass of femoral artery | L58 |
| Emergency bypass of femoral artery by anastomosis of femoral artery to femoral artery NEC | L58.1 |
| Emergency bypass of femoral artery by anastomosis of femoral artery to popliteal artery using prosthesis NEC | L58.2 |
| Emergency bypass of femoral artery by anastomosis of femoral artery to popliteal artery using vein graft NEC | L58.3 |
| Emergency bypass of femoral artery by anastomosis of femoral artery to tibial artery using prosthesis NEC | L58.4 |
| Emergency bypass of femoral artery by anastomosis of femoral artery to tibial artery using vein graft NEC | L58.5 |
| Emergency bypass of femoral artery by anastomosis of femoral artery to peroneal artery using prosthesis NEC | L58.6 |
| Emergency bypass of femoral artery by anastomosis of femoral artery to peroneal artery using vein graft NEC | L58.7 |
| Other specified other emergency bypass of femoral artery | L58.8 |
| Unspecified other emergency bypass of femoral artery | L58.9 |
| Other bypass of femoral artery | L59 |
| Bypass of femoral artery by anastomosis of femoral artery to femoral artery NEC | L59.1 |
| Bypass of femoral artery by anastomosis of femoral artery to popliteal artery using prosthesis NEC | L59.2 |
| Bypass of femoral artery by anastomosis of femoral artery to popliteal artery using vein graft NEC | L59.3 |
| Bypass of femoral artery by anastomosis of femoral artery to tibial artery using prosthesis NEC | L59.4 |
| Bypass of femoral artery by anastomosis of femoral artery to tibial artery using vein graft NEC | L59.5 |
| Bypass of femoral artery by anastomosis of femoral artery to peroneal artery using prosthesis NEC | L59.6 |
| Bypass of femoral artery by anastomosis of femoral artery to peroneal artery using vein graft NEC | L59.7 |
| Other specified other bypass of femoral artery | L59.8 |
| Unspecified other bypass of femoral artery | L59.9 |
| Reconstruction of femoral artery | L60 |
| Endarterectomy of femoral artery and patch repair of femoral artery | L60.1 |
| Endarterectomy of femoral artery NEC | L60.2 |
| Profundoplasty of femoral artery and patch repair of deep femoral artery | L60.3 |
| Profundoplasty of femoral artery NEC | L60.4 |
| Other specified reconstruction of femoral artery | L60.8 |
| Unspecified reconstruction of femoral artery | L60.9 |
| Other open operations on femoral artery | L62 |
| Repair of femoral artery NEC | L62.1 |
| Open embolectomy of femoral artery | L62.2 |
| Other specified other open operations on femoral artery | L62.8 |
| Unspecified other open operations on femoral artery | L62.9 |
| Percutaneous transluminal angioplasty of femoral artery | L63.1 |
| Percutaneous transluminal embolectomy of femoral artery | L63.2 |
| Percutaneous transluminal embolisation of femoral artery | L63.3 |
| Percutaneous transluminal insertion of stent into femoral artery | L63.5 |
| Revision of reconstruction of artery | L65 |
| Revision of reconstruction involving aorta | L65.1 |
| Revision of reconstruction involving iliac artery | L65.2 |
| Revision of reconstruction involving femoral artery | L65.3 |
| Transluminal balloon angioplasty of coronary artery | K49 |
| Percutaneous transluminal balloon angioplasty of one coronary artery | K49.1 |
| Percutaneous transluminal balloon angioplasty of multiple coronary arteries | K49.2 |
| Percutaneous transluminal balloon angioplasty of bypass graft of coronary artery | K49.3 |
| Percutaneous transluminal cutting balloon angioplasty of coronary artery | K49.4 |
| Other specified transluminal balloon angioplasty of coronary artery | K49.8 |
| Unspecified transluminal balloon angioplasty of coronary artery | K49.9 |
| Other therapeutic transluminal operations on coronary artery | K50 |
| Percutaneous transluminal laser coronary angioplasty | K50.1 |
| Percutaneous transluminal atherectomy of coronary artery | K50.4 |
| Other specified other therapeutic transluminal operations on coronary artery | K50.8 |
| Unspecified other therapeutic transluminal operations on coronary artery | K50.9 |
| Percutaneous transluminal balloon angioplasty and insertion of stent into coronary artery | K75 |
| Percutaneous transluminal balloon angioplasty and insertion of 1-2 drug-eluting stents into coronary artery | K75.1 |
| Percutaneous transluminal balloon angioplasty and insertion of 3 or more drug-eluting stents into coronary artery | K75.2 |
| Percutaneous transluminal balloon angioplasty and insertion of 1-2 stents into coronary artery | K75.3 |
| Percutaneous transluminal balloon angioplasty and insertion of 3 or more stents into coronary artery NEC | K75.4 |
| Other specified percutaneous transluminal balloon angioplasty and insertion of stent into coronary artery | K75.8 |
| Unspecified percutaneous transluminal balloon angioplasty and insertion of stent into coronary artery | K75.9 |
| External resuscitation | X50 |
| Advanced cardiac pulmonary resuscitation | X503 |
| External ventricular defibrillation | X504 |
| Other specified external resuscitation | X508 |
| Unspecified external resuscitation | X509 |

Supplementary Table 2 – Patient characteristics in validation dataset, *n* (%) unless specified

|  | Total |
| --- | --- |
| *n* | 226,024 |
| Age: Mean (SD) | 47.3 (16.6) |
| Sex: Female | 120,046 (53.1) |
| Ethnicity: |  |
| White/Not recorded | 210201 (93.0) |
| Asian | 8761 (3.9) |
| Black | 3997 (1.8) |
| Other | 3065 (1.4) |
| Deprivation: |  |
| Least | 55028 (24.3) |
| 2^nd^ | 51712 (22.9) |
| 3^rd^ | 46336 (20.5) |
| 4^th^ | 43484 (19.2) |
| Most | 29464 (13.0) |
| Smoking status |  |
| Never | 107411 (47.5) |
| Current | 65870 (29.1) |
| Ex | 46721 (20.7) |
| Not recorded | 6022 (2.7) |
| Diabetes type 1 | 372 (0.2) |
| Diabetes type 2 | 8881 (3.9) |
| Family history of CVD | 6436 (2.8) |
| CKD stage 3-5 | 8136 (3.6) |
| Atrial fibrillation | 2224 (1.0) |
| Treated hypertension | 45088 (19.9) |
| Migraine | 6341 (2.8) |
| Rheumatoid arthritis | 1031 (0.5) |
| Severe mental illness | 4526 (2.0) |
| Corticosteroid medication | 11431 (5.1) |
| Cholesterol/HDL ratio: Mean (SD) | 3.9 (0.9) |
| BMI: |  |
| Underweight | 82858 (36.7) |
| Overweight | 66087 (29.2) |
| Obese | 45193 (20.0) |
| Not recorded | 31886 (14.1) |
| Depression/anxiety | 33366 (14.8) |
| Oesophageal reflux | 22231 (9.8) |
| Respiratory | 46707 (20.7) |
| Osteoarthritis | 8143 (3.6) |
| Low back pain | 42591 (18.8) |
| Neck pain | 17496 (7.7) |
| Cancer | 4265 (1.9) |
| CVD in follow-up | 21,495 (9.5)  18.4 /1000py |

BMI: Body mass index; CVD: Cardiovascular disease; SD: Standard deviation; py: person-years. Missing data cholesterol 54%, ethnicity 10%

Supplementary Table 3 – Associations with future cardiovascular disease in females and in males with unattributed chest pain in development dataset

|  | Males |  |  |  | Females |  |  |  |
| --- | --- | --- | --- | --- | --- | --- | --- | --- |
|  | No CVD | CVD | Unadjusted  HR (95% CI) | Adjusted  HR (95% CI) | No CVD | CVD | Unadjusted  HR (95% CI) | Adjusted  HR (95% CI) |
| *n* | 153778 | 21532 |  |  | 178188 | 21419 |  |  |
| Age: Mean (SD) | 44.6 (15.3) | 60.2 (13.7) | 1.07 (1.07, 1.07) | 1.06 (1.06, 1.07) | 47.1 (16.31) | 63.7 (14.30) | 1.07 (1.07, 1.07) | 1.06 (1.06, 1.06) |
| Ethnicity |  |  |  |  |  |  |  |  |
| White/Not stated | 136726 (88.9) | 19889 (92.4) | 1 | 1 | 156406 (87.8) | 19804 (92.5) | 1 | 1 |
| Asian | 9012 (5.9) | 1048 (4.9) | 0.95 (0.88, 1.02) | 1.22 (1.13, 1.31) | 10863 (6.1) | 918 (4.3) | 0.77 (0.71, 0.84) | 1.10 (1.02, 1.19) |
| Black | 5054 (3.3) | 418 (1.9) | 0.71 (0.63, 0.79) | 0.70 (0.63, 0.78) | 7741 (4.3) | 556 (2.6) | 0.71 (0.64, 0.78) | 0.84 (0.76, 0.93) |
| Other | 2986 (1.9) | 177 (0.8) | 0.55 (0.48, 0.63) | 0.86 (0.75, 1.00) | 3178 (1.8) | 141 (0.7) | 0.48 (0.41, 0.57) | 0.87 (0.74, 1.03) |
| Deprivation |  |  |  |  |  |  |  |  |
| Least | 33616 (21.9) | 4865 (22.6) | 1 | 1 | 38367 (21.5) | 4415 (20.6) | 1 | 1 |
| 2^nd^ | 30620 (19.9) | 4592 (21.3) | 1.05 (1.00, 1.10) | 1.03 (0.99, 1.08) | 35553 (20.0) | 4502 (21.0) | 1.12 (1.06, 1.17) | 1.08 (1.03, 1.14) |
| 3^rd^ | 29159 (19.0) | 4237 (19.7) | 1.05 (1.00, 1.11) | 1.12 (1.07, 1.18) | 33704 (18.9) | 4253 (19.9) | 1.13 (1.07, 1.20) | 1.13 (1.07, 1.19) |
| 4^th^ | 27862 (18.1) | 3728 (17.3) | 1.03 (0.98, 1.09) | 1.18 (1.12, 1.25) | 31943 (17.9) | 3915 (18.3) | 1.14 (1.08, 1.21) | 1.23 (1.16, 1.30) |
| Most | 32521 (21.1) | 4110 (19.1) | 1.04 (0.98, 1.10) | 1.31 (1.23, 1.39) | 38621 (21.7) | 4334 (20.2) | 1.11 (1.05, 1.19) | 1.35 (1.26, 1.43) |
| Smoking status |  |  |  |  |  |  |  |  |
| Never | 64522 (42.0) | 7387 (34.3) | 1 | 1 | 91334 (51.3) | 9883 (46.1) | 1 | 1 |
| Current | 46934 (30.5) | 6355 (29.5) | 1.15 (1.10, 1.19) | 1.48 (1.43, 1.54) | 46735 (26.2) | 5714 (26.7) | 1.06 (1.02, 1.10) | 1.42 (1.37, 1.48) |
| Ex | 36141 (23.5) | 7134 (33.1) | 1.69 (1.63, 1.76) | 1.10 (1.07, 1.14) | 37055 (20.8) | 5291 (24.7) | 1.31 (1.26, 1.37) | 1.11 (1.07, 1.15) |
| Not recorded | 6181 (4.0) | 656 (3.0) | 0.63 (0.58, 0.69) | 1.15 (1.05, 1.25) | 3064 (1.7) | 531 (2.5) | 1.00 (0.91, 1.09) | 1.20 (1.09, 1.32) |
| Diabetes type 1 | 578 (0.4) | 144 (0.7) | 1.85 (1.56, 2.19) | 2.23 (1.87, 2.66) | 501 (0.3) | 122 (0.6) | 2.22 (1.84, 2.69) | 2.68 (2.17, 3.32) |
| Diabetes type 2 | 7885 (5.1) | 2428 (11.3) | 2.72 (2.60, 2.85) | 1.21 (1.16, 1.28) | 7889 (4.4) | 2242 (10.5) | 2.95 (2.80, 3.10) | 1.44 (1.36, 1.52) |
| FH: angina/heart attack <60yrs | 7351 (4.8) | 1246 (5.8) | 1.16 (1.10, 1.24) | 1.27 (1.19, 1.34) | 9218 (5.2) | 1265 (5.9) | 1.13 (1.06, 1.20) | 1.21 (1.14, 1.28) |
| CKD stage 3-5 | 4864 (3.2) | 1783 (8.3) | 3.53 (3.35, 3.71) | 0.98 (0.93, 1.04) | 9698 (5.4) | 2938 (13.7) | 3.43 (3.27, 3.59) | 1.02 (0.98, 1.07) |
| Atrial fibrillation | 1556 (1.0) | 1048 (4.9) | 4.91 (4.59, 5.26) | 1.75 (1.63, 1.88) | 1197 (0.7) | 970 (4.5) | 6.82 (6.37, 7.30) | 2.10 (1.95, 2.26) |
| Treated hypertension | 24045 (15.6) | 8446 (39.2) | 3.47 (3.36, 3.59) | 1.49 (1.43, 1.55) | 34506 (19.4) | 10642 (49.7) | 3.90 (3.76, 4.04) | 1.59 (1.54, 1.66) |
| Migraine | 2178 (1.4) | 267 (1.2) | 0.92 (0.81, 1.03) | 1.10 (0.97, 1.24) | 8090 (4.5) | 642 (3.0) | 0.70 (0.64, 0.76) | 1.12 (1.03, 1.21) |
| Rheumatoid arthritis | 456 (0.3) | 160 (0.7) | 2.78 (2.38, 3.25) | 1.24 (1.06, 1.44) | 1404 (0.8) | 342 (1.6) | 2.28 (2.06, 2.53) | 1.29 (1.17, 1.43) |
| Severe mental illness | 2501 (1.6) | 412 (1.9) | 1.26 (1.14, 1.38) | 1.25 (1.13, 1.38) | 3482 (2.0) | 573 (2.7) | 1.32 (1.21, 1.44) | 1.29 (1.18, 1.41) |
| Corticosteroid medication | 5339 (3.5) | 1579 (7.3) | 2.40 (2.27, 2.53) | 1.27 (1.20, 1.35) | 10652 (6.0) | 2580 (12.0) | 2.28 (2.19, 2.38) | 1.31 (1.25, 1.37) |
| Cholesterol/HDL ratio: Mean (SD) | 4.3 (1.04) | 4.2 (1.12) | 0.91 (0.89, 0.93) | 1.08 (1.07, 1.09) | 3.6 (0.88) | 3.7 (0.96) | 1.06 (1.05, 1.08) | 1.06 (1.05, 1.08) |
| Body mass index |  |  |  |  |  |  |  |  |
| Underweight | 46519 (30.3) | 5441 (25.3) | 1 | 1 | 71343 (40.0) | 6727 (31.4) | 1 | 1 |
| Overweight | 48280 (31.4) | 7730 (35.9) | 1.32 (1.27, 1.36) | 1.02 (0.99, 1.06) | 47042 (26.4) | 6351 (29.7) | 1.40 (1.35, 1.45) | 1.05 (1.01, 1.09) |
| Obese | 27488 (17.9) | 5254 (24.4) | 1.62 (1.55, 1.68) | 1.27 (1.22, 1.32) | 41490 (23.3) | 5966 (27.9) | 1.54 (1.48, 1.61) | 1.19 (1.14, 1.24) |
| Not recorded | 31491 (20.5) | 3107 (14.4) | 0.70 (0.66, 0.74) | 1.03 (0.98, 1.09) | 18313 (10.3) | 2375 (11.1) | 1.06 (0.99, 1.13) | 1.13 (1.06, 1.21) |
| Depression/anxiety | 18892 (12.3) | 2367 (11.0) | 0.97 (0.93, 1.01) | 1.13 (1.08, 1.18) | 36267 (20.4) | 4114 (19.2) | 0.96 (0.92, 0.99) | 1.15 (1.11, 1.19) |
| Oesophageal reflux | 12666 (8.2) | 2344 (10.9) | 1.32 (1.27, 1.37) | 1.07 (1.02, 1.11) | 17265 (9.7) | 2820 (13.2) | 1.36 (1.30, 1.42) | 1.06 (1.02, 1.11) |
| Respiratory | 26633 (17.3) | 5231 (24.3) | 1.52 (1.47, 1.58) | 1.13 (1.09, 1.17) | 40139 (22.5) | 6749 (31.5) | 1.55 (1.50, 1.60) | 1.21 (1.17, 1.25) |
| Osteoarthritis | 4440 (2.9) | 1667 (7.7) | 2.62 (2.48, 2.76) | 0.99 (0.93, 1.04) | 9495 (5.3) | 2823 (13.2) | 2.51 (2.41, 2.63) | 1.04 (0.99, 1.08) |
| Low back pain | 23127 (15.0) | 3603 (16.7) | 1.15 (1.10, 1.19) | 1.04 (1.00, 1.08) | 33386 (18.7) | 4600 (21.5) | 1.20 (1.16, 1.24) | 1.10 (1.06, 1.14) |
| Neck pain | 8340 (5.4) | 1597 (7.4) | 1.32 (1.25, 1.39) | 1.11 (1.05, 1.17) | 13993 (7.9) | 1927 (9.0) | 1.09 (1 .04, 1.14) | 1.02 (0.97, 1.07) |
| Cancer | 3258 (2.1) | 941 (4.4) | 2.97 (2.78, 3.17) | 1.09 (1.02, 1.17) | 4486 (2.5) | 836 (3.9) | 2.03 (1.90, 2.17) | 1.16 (1.08, 1.24) |

Supplementary Table 4 – Internal and external validation for gender-specific models

|  | Males | Females |
| --- | --- | --- |
| ***Internal validation*** |  |  |
| C-Statistic (95% CI) | 0.793 (0.790, 0.796) | 0.799 (0.796, 0.802) |
| D Statistic (Standard error) | 1.833 (0.012) | 1.925 (0.012) |
| R-squared | 0.444 | 0.469 |
| Calibration slope | 1 (0.984, 1.016) | 1 (0.982, 1.018) |
| E/O event probabilities at 10 years | 1.008 | 1.009 |
| Heuristic shrinkage factor | 0.996 | 0.997 |
| ***External validation*** |  |  |
| C-Statistic (95% CI) | 0.801 (0.797, 0.805) | 0.806 (0.802, 0.811) |
| Calibration slope | 1.008 (0.986, 1.031) | 1.029 (1.001, 1.057) |
| E/O event probabilities at 10 years | 0.999 | 1.000 |

Supplementary Table 5 – Coefficients for developed models

|  | Model 5 | Model 2 |
| --- | --- | --- |
| Baseline survival at 10 years | 0.9128141 | 0.9154936 |
| Covariate | Coefficient | Coefficient |
| Age - 47.76824 |  | 0.0604704 |
| ln(age/10) - 1.563775822 | 3.164345 |  |
| (age/10)^3 - 108.9977745 | 0.0004508 |  |
| Ethnicity |  |  |
| White/Not stated | 0 | 0 |
| Asian | 0.1954782 | 0.1525606 |
| Black | -0.3587095 | -0.3063235 |
| Other | -0.1445255 | -0.161262 |
| Deprivation |  |  |
| Least deprived | 0 | 0 |
| 2^nd^ least deprived | 0.0580011 | 0.0644697 |
| Mid deprived | 0.1232874 | 0.131571 |
| 2^nd^ most deprived | 0.1945437 | 0.2057404 |
| Most deprived | 0.2916882 | 0.3127611 |
| Sex |  |  |
| Male | 0 | 0 |
| Female | -0.4209539 | -0.3460529 |
| Smoking status |  |  |
| Never | 0 | 0 |
| Current | 0.365312 | 0.4053198 |
| Ex | 0.1007734 | 0.1267552 |
| Missing | 0.179496 | 0.1463052 |
| Type 1 diabetes | 0.8784455 | 0.8462363 |
| Type 2 diabetes | 0.1939225 | 0.2612422 |
| Family history of CVD | 0.1839979 | 0.2073142 |
| Chronic kidney disease | 0.0417059 | 0.0244556 |
| Atrial fibrillation | 0.5895643 | 0.6397064 |
| Hypertension | 0.3991172 | 0.4362309 |
| Migraine | 0.1019881 |  |
| Rheumatoid arthritis | 0.2338624 |  |
| Severe mental illness | 0.2306164 |  |
| Corticosteroid use | 0.262343 |  |
| Body mass index |  |  |
| Normal weight | 0 | 0 |
| Overweight | -0.0111599 | 0.0575463 |
| Obese | 0.1814732 | 0.256817 |
| Not recorded | 0.0326476 | 0.0717883 |
| Depression, anxiety, or stress | 0.1250154 |  |
| Oesophageal reflux | 0.0584708 |  |
| Respiratory conditions | 0.1243584 |  |
| Low back pain | 0.0676378 |  |
| Neck pain | 0.0542081 |  |
| Cancer | 0.1323498 |  |
| (Total cholesterol to HDL ratio/10)^2-.1528495691 | 1.486199 |  |
| (Total cholesterol to HDL ratio/10)^3-.0597580377 | -0.6544413 |  |
| Female * (ln(age/10) - 1.563775822) | -0.5908441 |  |
| Female * ((age/10)^3 - 108.9977745) | 0.0007694 |  |
| Female * Asian ethnicity | -0.1028719 |  |
| Female * Black ethnicity | 0.1885113 |  |
| Female * Other ethnicity | 0.0147175 |  |
| Female * type 2 diabetes | 0.1642018 |  |
| Female * atrial fibrillation | 0.1667749 |  |
| Female * hypertension | 0.0727926 |  |
| Female * overweight | 0.0488138 |  |
| Female * obese | -0.0270129 |  |
| Female * no recorded BMI | 0.0981893 |  |
| Female * respiratory conditions | 0.0697061 |  |

Supplementary Figure 1 – Calibration plots of QRISK3 score in development and validation datasets

| Development dataset  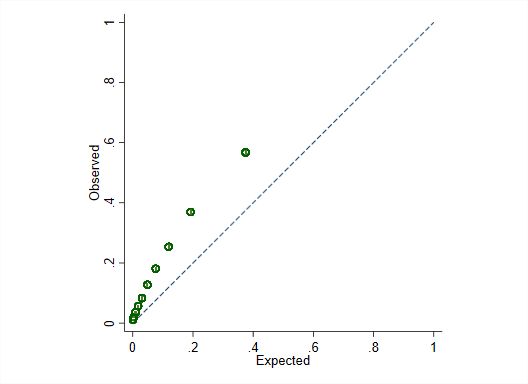  C statistic: 0.789 (0.787, 0.791)  C slope: 0.752 (0.744, 0.760) | Validation dataset  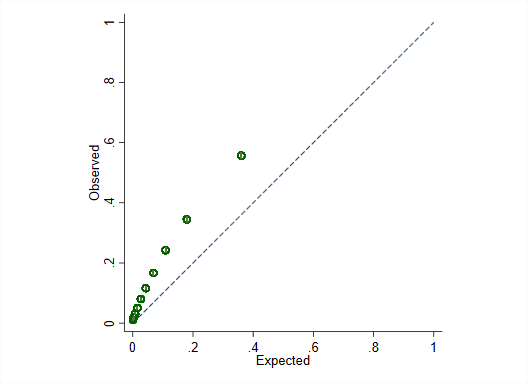  C statistic: 0.798 (0.795, 0.801)  C slope: 0.767 (0.747, 0.786) |
| --- | --- |

Supplementary Figure 2 – Calibration plots for full model (model 5) by deprivation in validation dataset

| LEAST DEPRIVED (1)  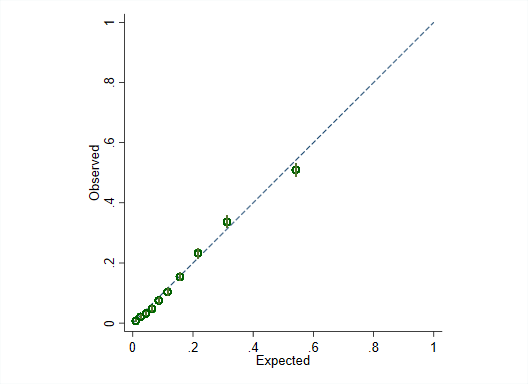  C statistic: 0.806 (0.800, 0.812)  C slope: 1.085 (1.044, 1.126) | 2^ND^ LEAST DEPRIVED (2)  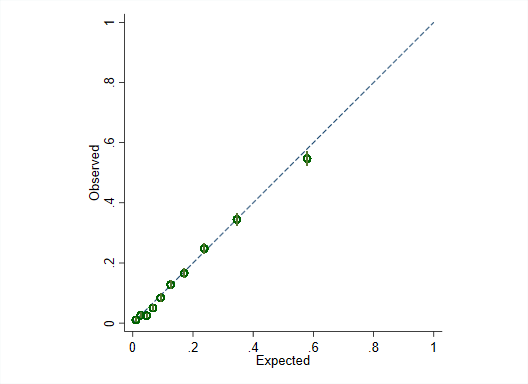  C statistic: 0.802 (0.797, 0.808)  C slope: 1.044 (1.008, 1.080) |
| --- | --- |
| MID-DEPRIVED (3)  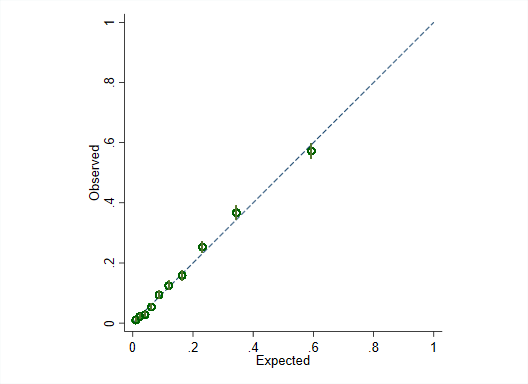  C statistic: 0.807 (0.800, 0.813)  C slope: 1.023 (0.990, 1.056) | 2^ND^ MOST DEPRIVED (4)  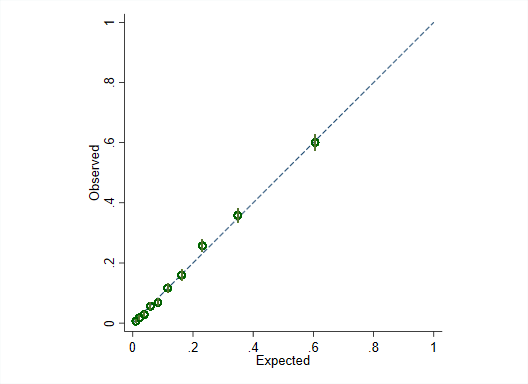  C statistic: 0.809 (0.803, 0.816)  C slope: 1.008 (0.970, 1.046) |
| MOST DEPRIVED (5)  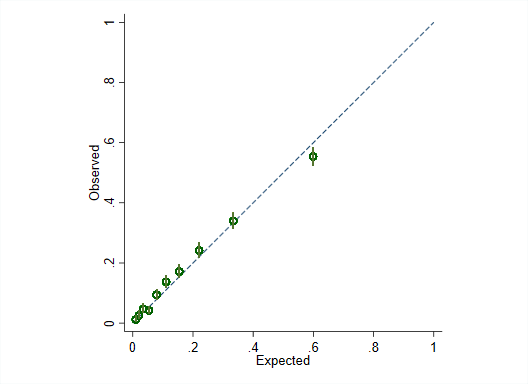  C statistic: 0.795 (0.788, 0.803)  C slope: 0.913 (0.862, 0.964) |  |

Supplementary Figure 3 – Calibration plots for full model (model 5) by geographical region in validation dataset

| REGION (1)  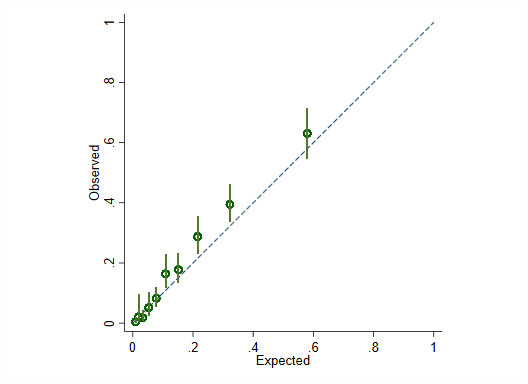  C statistic: 0.810 (0.794, 0.827)  C slope: 1.024 (0.931, 1.116) | REGION (2)  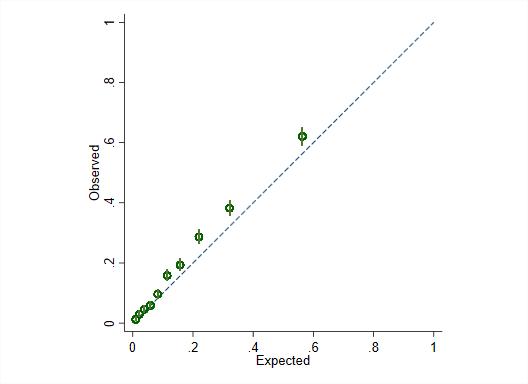  C statistic: 0.802 (0.795, 0.809)  C slope: 0.999 (0.949, 1.049) |
| --- | --- |
| REGION (3)  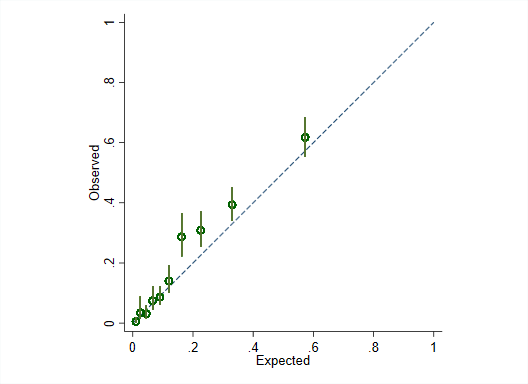  C statistic: 0.797 (0.783, 0.812)  C slope: 0.990 (0.917, 1.064) | REGION (4)  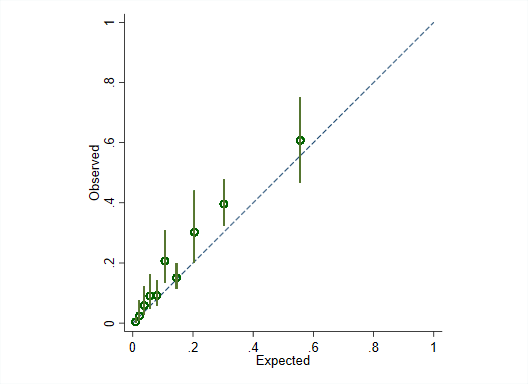  C statistic: 0.786 (0.768, 0.805)  C slope: 0.942 (0.748, 1.135) |
| REGION (5)  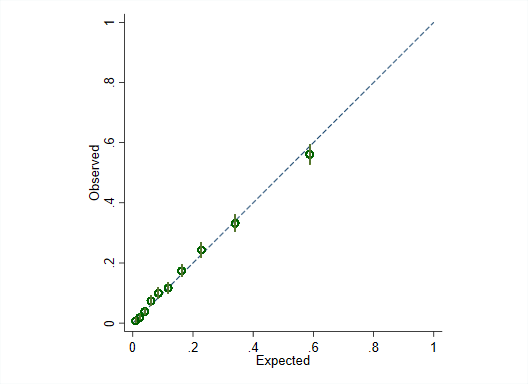  C statistic: 0.791 (0.783, 0.799)  C slope: 0.953 (0.870, 1.036) | REGION (6)  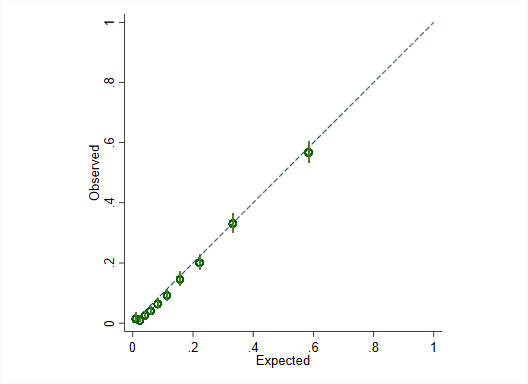  C statistic: 0.824 (0.816, 0.832)  C slope: 1.084 (1.015, 1.152) |
| REGION (7)  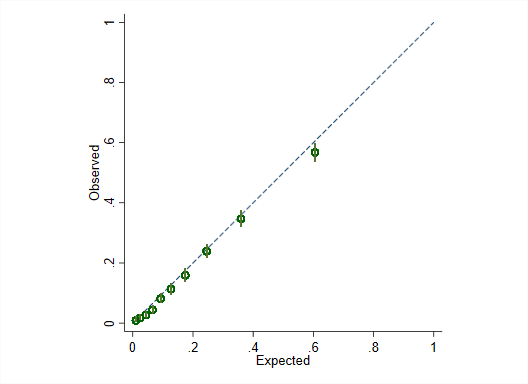  C statistic: 0.817 (0.810, 0.824)  C slope: 1.077 (1.031, 1.123) | REGION (8)  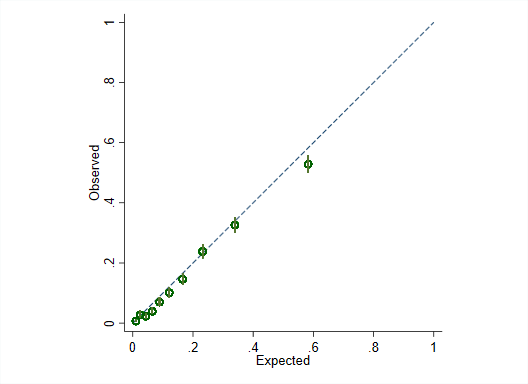  C statistic: 0.809 (0.801, 0.817)  C slope: 1.066 (1.013, 1.118) |
| REGION (9)  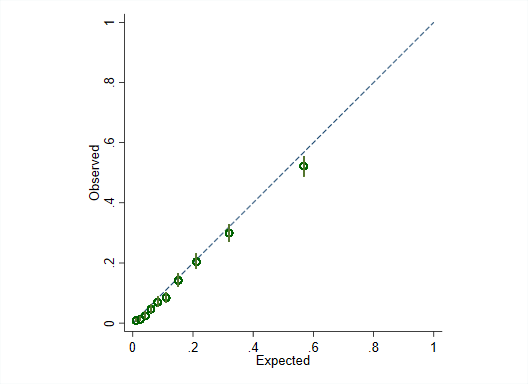  C statistic: 0.802 (0.792, 0.811)  C slope: 1.031 (0.940, 1.122) | REGION (10)  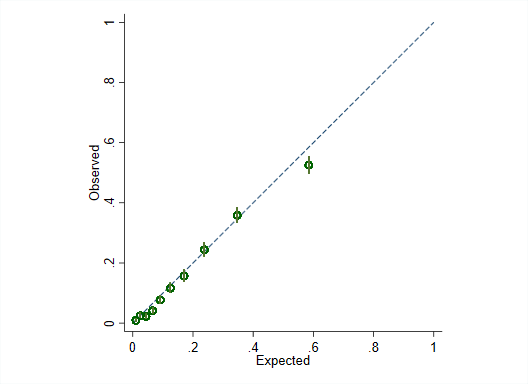  C statistic: 0.802 (0.795, 0.809)  C slope: 1.035 (0.992, 1.079) |
